# Benzimidazole carbamate induces cytotoxicity in breast cancer cells via two distinct cell death mechanisms

**DOI:** 10.1038/s41420-023-01454-6

**Published:** 2023-05-13

**Authors:** Brendan T. Graff, Chitra Palanivel, Christopher B. Jenkins, Janina Baranowska-Kortylewicz, Ying Yan

**Affiliations:** 1 Department of Radiation Oncology, College of Medicine University of Nebraska Medical Center Omaha, Nebraska, USA; 2 Department of Pharmaceutical Sciences, College of Pharmacy University of Nebraska Medical Center Omaha, Nebraska, USA; 3 Department of Biochemistry and Molecular Biology, College of Medicine University of Nebraska Medical Center Omaha, Nebraska, USA

**Keywords:** Breast cancer, Cytoskeleton, Cell death

## Abstract

Metastatic breast cancer (mBC) is responsible for >90% of breast cancer-related deaths. Microtubule-targeting agents (MTAs) are the front-line treatment for mBC. However, the effectiveness of MTAs is frequently limited by the primary or acquired resistance. Furthermore, recurrent mBC derived from cancer cells that survived MTA treatment are typically more chemoresistant. The overall response rates for the second- and third-line MTAs in mBC patients previously treated with MTAs are 12–35%. Thus, there is an ongoing search for novel MTAs with a distinct mode of action that can circumvent chemoresistance mechanisms. Our results show that *methyl N-(6-benzoyl-1H-****b****enzimidazol-2-yl)****car****bamate* (BCar), a microtubule-disrupting anthelmintic that binds to the colchicine binding site separate from the binding sites of clinically used MTAs, has the potential to treat MTA-resistant mBC. We have comprehensively evaluated the cellular effects of BCar in a panel of human breast cancer (BC) cell lines and normal breast cells. BCar effects on the clonogenic survival, cell cycle, apoptosis, autophagy, senescence, and mitotic catastrophe were measured. Approximately 25% of BCs harbor mutant p53. For this reason, the p53 status was included as a variable. The results show that BC cells are >10x more sensitive to BCar than normal mammary epithelial cells (HME). p53-mutant BC cells are significantly more sensitive to BCar treatment than p53 wild-type BC cells. Furthermore, BCar appears to kill BC cells primarily via either p53-dependent apoptosis or p53-independent mitotic catastrophe. When compared to docetaxel and vincristine, two clinical MTAs, BCar is fairly innocuous in HME cells, providing a much wider therapeutic window than docetaxel and vincristine. Together, the results strongly support the notion that BCar-based therapeutics may serve as a new line of MTAs for mBC treatment.

## Introduction

Breast cancer (BC) remains the second leading cause of cancer-related deaths in the US. Nearly 90% of deaths are attributable to metastatic breast cancer (mBC) [1]. mBC is typically treated with chemotherapy, which includes endocrine therapy, DNA-alkylating agents, microtubule-targeting agents, and more recently, antibody-drug conjugates [2, 3]. Microtubule-targeting agents (MTAs) remain the standard of care for mBC treatment [4]. Most often utilized MTAs include paclitaxel, a microtubule-stabilizing agent, and vincristine, a microtubule-destabilizing agent used to treat advanced-stage BC [4–6]. However, chemoresistance, primary or acquired, and various side effects diminish the efficacy of current MTAs [4–6]. Furthermore, the failure of the first round of MTA treatment often negatively impacts the response to the second and third-line MTAs. The overall response rates of the subsequent MTA treatments range from 12% to 35% [7–12]. Thus, there is a significant need to develop new MTAs that are less toxic and have distinct mechanisms of action (MOA).

**Fig. 1:**
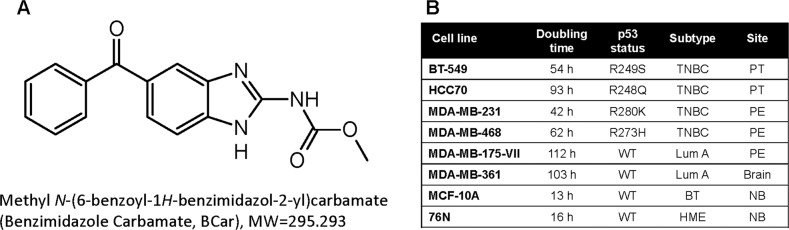
Chemical structure of BCar and breast cancer cell line subtypes. **A** Chemical structure of methyl N-(6-benzoyl-1H-benzimidazol-2-yl)carbamate (BCar). **B** Characteristics of the human normal mammary epithelial cells and breast cancer cell lines, which were used in the study (https://www.atcc.org/) [36, 37]. PT primary tumor, PE pleural effusion, BT benign tumor, Lum luminal, TNBC triple-negative breast cancer, WT wild type, NB normal breast, HME human mammary epithelial.

Clinical MTAs bind to tubulin either via the vinca- or taxane-binding sites [4, 6]. Evaluated in this study methyl *N*-(6-benzoyl-1*H*-benzimidazol-2-yl)carbamate, the microtubule depolymerizing compound (BCar; Fig. 1), binds to the colchicine binding site remote from the vinca and taxane sites and located at the interface between α- and β-tubulin [13]. The binding of BCar to this site impedes the dimerization of α- and β-tubulin [13, 14]. BCar is an FDA-approved anti-parasitic oral drug with a clinically proven favorable safety profile [15–18]. In search for novel anti-cancer MTAs, BCar and related albendazole and flubendazole [19, 20] have been repurposed for the treatment of various malignancies [15, 21–23]. The cytotoxicity of BCar and related compounds in cancer cells is uniformly attributed to their ability to depolymerize microtubules [24–26]. However, the underlying cell death mechanisms remain mostly undefined. The cytotoxic effects of BCar in normal cells, an essential measure of the drug’s clinical potential, has yet to be characterize.

Tumor suppressor p53 plays an essential role in suppressing oncogenesis via the induction and coordination of DNA damage checkpoint responses, to promote DNA damage repair and cell survival [27]. However, if the DNA damage is not repairable, p53 will induce either apoptosis or senescence to protect the genome stability [27]. The loss of p53 function via mutations is the most frequent event in human malignancies [27]. For instance, in difficult-to-treat triple-negative breast cancer (TNBC), mutant p53 is detected in >80% of cases [28, 29]. Emerging data also suggest that the loss of the p53 function is a key driver of cancer progression and metastasis [30].

Mutations in p53 are associated with BC sensitivity to clinical MTAs [31, 32], whereas clinical responses of BC to chemotherapy have no direct relationship to the classical p53-dependent apoptosis, a pattern also observed in other cancer cell models [33]. For example, estrogen receptor-positive BCs, predominantly with wild-type (wt) p53, are often resistant to chemotherapy, whereas estrogen receptor-negative BCs, frequently with p53 mutations are more chemo-sensitive [34, 35].

The current study evaluated BCar’s cytotoxicity in BC cells and human normal mammary epithelial (HME) cells and elucidated mechanisms of BCar-induced cell death.

## Results

### Breast cancer cells are more sensitive to BCar than normal mammary epithelial cells

**Fig. 2:**
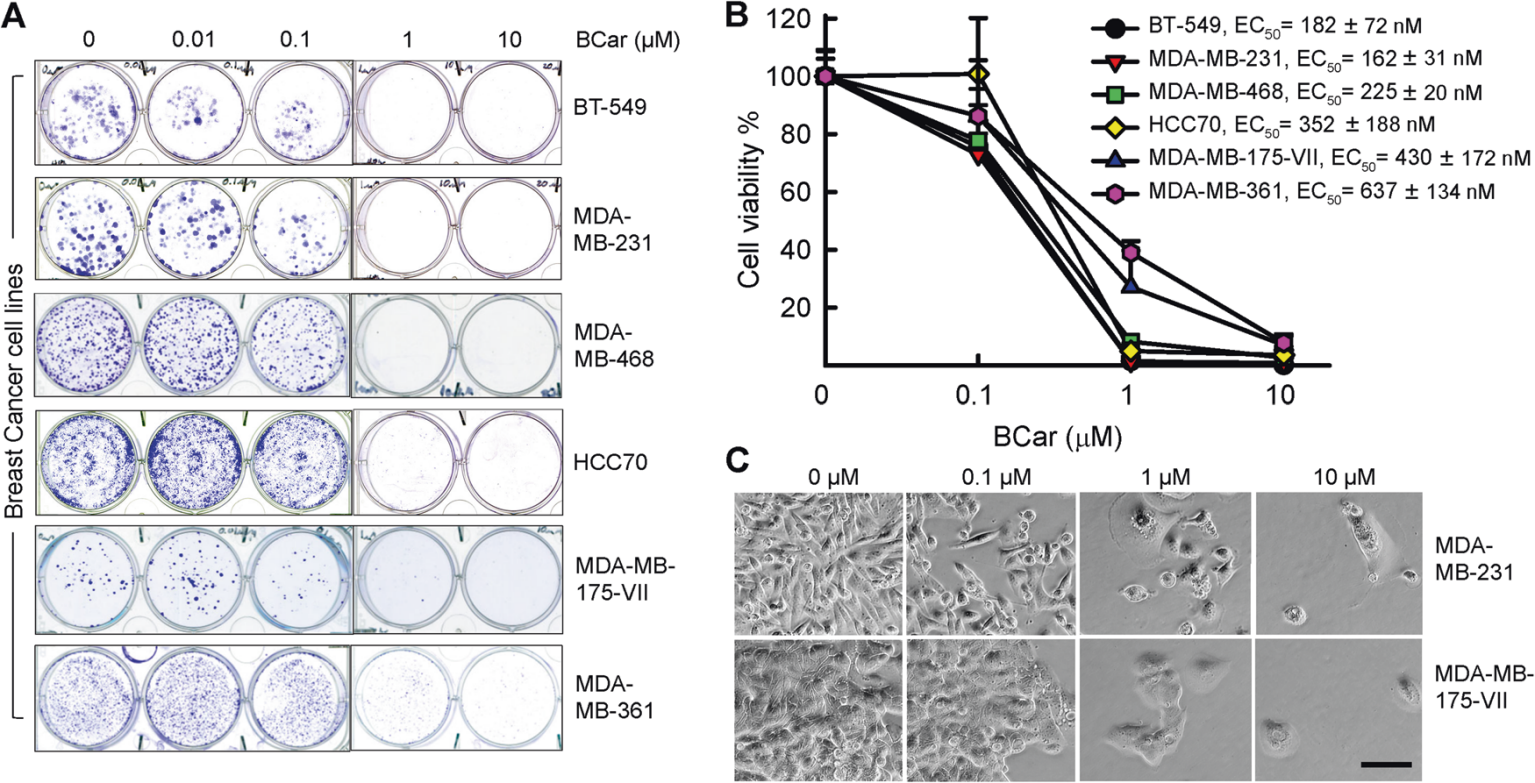
The effect of BCar on the clonogenic survival of breast cancer cells. Log-phase growing breast cancer cells were incubated with BCar at the indicated concentrations for 48 h, washed, and then incubated in a fresh growth medium for two weeks. Colonies were visualized by the crystal violet staining. **A** Representative samples from clonogenic survival assays. **B** Colonies in the resulting samples were quantified using ImageJ and analyzed using SigmaPlot software. Results are shown as mean ± s.d. of two sets of experiments in duplicate samples. EC_50_ values were determined using nonlinear regression with GraphPad Prism software. **C** Cells morphology photographed using phase-contrast optics. Scale bars represent 100 µm.

**Fig. 3:**
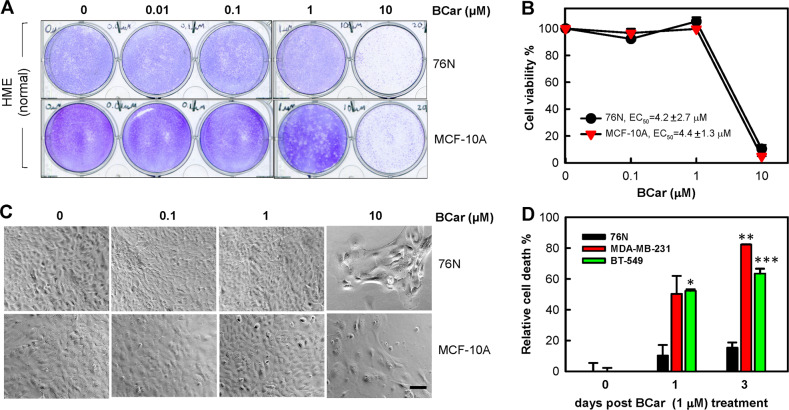
The effect of BCar on the viability of human normal mammary epithelial cells (HME) and breast cancer cells. Log-phase growing HME cells were incubated with BCar for 48 h at the indicated concentrations, washed, and incubated in a growth medium for 14 days. The surviving cells were visualized by crystal violet staining. **A** Representative samples from cell viability assay. **B** Cell survival in the resulting samples was quantified using ImageJ and the results are shown as mean ± s.d. of two sets of experiments in duplicate samples. EC_50_ values were determined using nonlinear regression with GraphPad Prism software. **C** Cells were photographed using phase-contrast optics. Scale bars represent 100 µm. **D** HME and breast cancer cells were incubated with 1 µM BCar for the specified time. Dead cells were quantified by the trypan blue exclusion assay. Results are shown as mean ± s.d. of two sets of experiments in duplicate samples.

The dose-dependent effect of BCar on cell viability was evaluated in a panel of breast normal and malignant cell lines. Since ~25% of BCs express mutant p53 (p53-mt), we also compared the survival of BCar-treated BC cells expressing p53-wt to BC cells with p53-mt. The p53 mutational status and characteristics of these breast cell lines are shown in Fig. 1B (https://www.atcc.org) [36, 37]. BCar treatment resulted in dose-dependent inhibition of the clonogenic viability in all tested breast cancer cell lines, with EC_50_ ranging from 162 nM to 637 nM (Fig. 2). Furthermore, p53-mt breast cancer cells (*BT-549, MDA-MB-231*, *MDA-MB-468*, and *HCC70*) were more sensitive to BCar treatment compared to p53-wt breast cancer cells (MDA-MB-175-VII and MDA-MB-361) (Fig. 2 and Table 1). In contrast, BCar EC_50_ of 4.2 µM and 4.4 µM was measured in human normal (76 N) and non-tumorigenic benign (MCF10A) mammary epithelial (HME) cells, respectively, i.e., HMEs appear to be ~10 times more resistant to BCar treatment than BC cell lines (Fig. 3 and Table 1). To validate the differential cytotoxic effects of BCar in breast normal and cancer cells, we compared early cell death response following BCar treatment of HME and BC cells using trypan blue exclusion assay. As shown in Fig. 3D, within 3 days of BCar treatment, BC cells died at a much higher rate than HME cells.

**Table 1:** BCar EC_50_ Values in breast normal and malignant cell lines were compared using unpaired *t* test. The *P* values showing significant differences (<0.05) are bolded.

Cell line	EC50 [nM]	S.D. (*n* = 6)	*P* values MCF-10A vs. other	*P* values 76 N vs. other	*P* values BT-549 vs. other	*P* values HCC70 vs. other	*P* values MDA-MB-468 vs. other	*P* values MDA-MB-231 vs. other	*P* values MDA-MB-361 vs. other
**MCF-10A**	4409	1325							
**76N**	4156	2733	0.9999						
**BT-549**	182	72	**<0.0001**	**<0.0001**					
**HCC70**	352	188	**<0.0001**	**<0.0001**	0.1830				
**MDA-MB-468**	225	20	**<0.0001**	**<0.0001**	0.9160	0.7144			
**MDA-MB-231**	162	31	**<0.0001**	**<0.0001**	0.9997	0.1049	0.7939		
**MDA-MB-361**	637	134	**<0.0001**	**0.0001**	**<0.0001**	**0.0041**	**0.0001**	**<0.0001**	
**MDA-MB-175-VII**	430	231	**<0.0001**	**<0.0001**	**0.0158**	0.8741	0.1476	**0.0077**	0.0626

### Effects of BCar on cell cycle response of normal and malignant breast cells

**Fig. 4:**
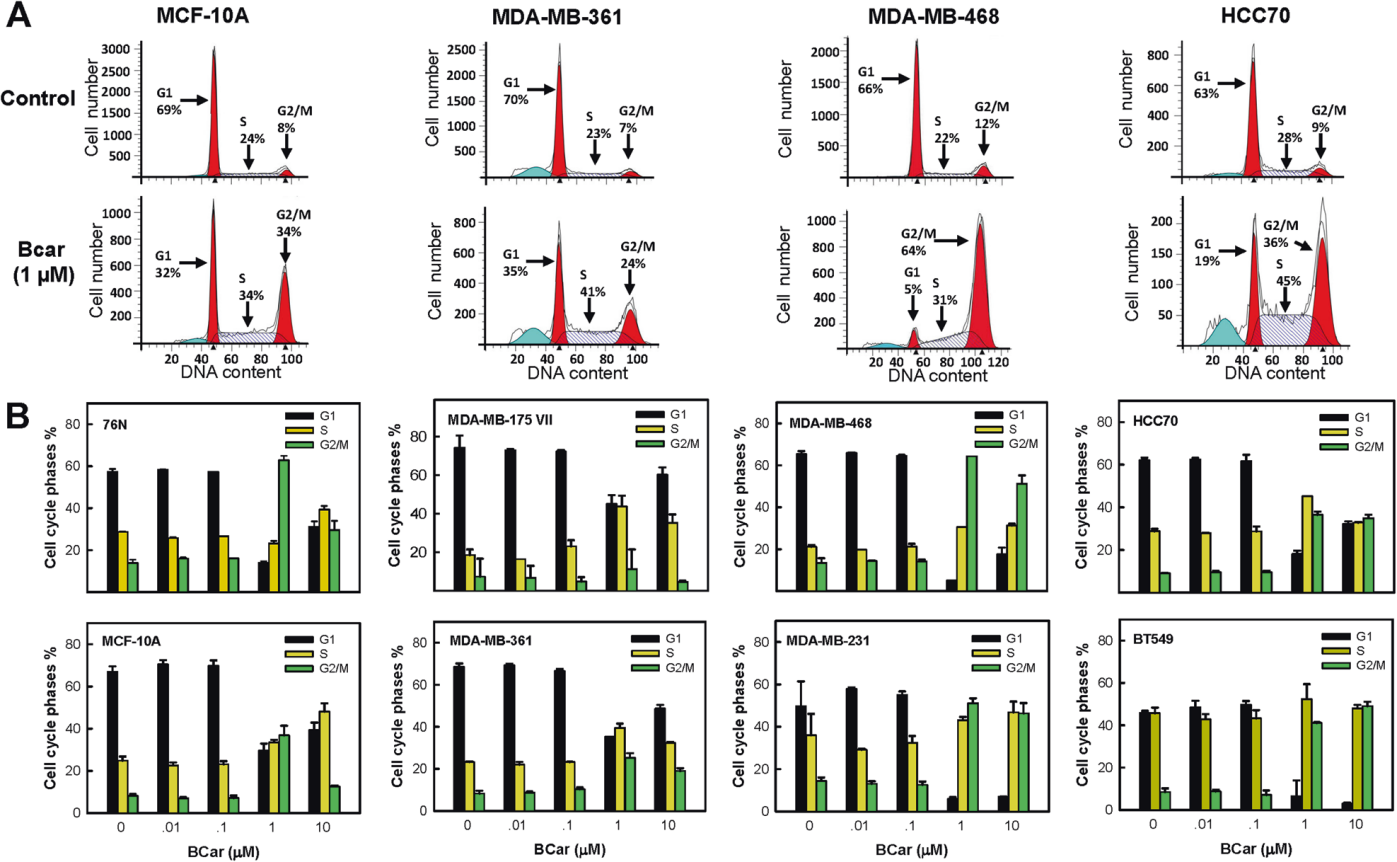
Effect of BCar on cell cycle response of human breast normal and cancer cells. Log-phase growing cells were incubated with BCar at the indicated concentrations for 48 h and analyzed for DNA content by FACS. **A** Representative diagrams display the cell cycle profiles of the control treated or BCar (1 µM) treated cells. The percentages of cells in the G1, S, and G2/M phases of the cell cycle are indicated. **B** Bar graphs depict the percentage of cells in the G1, S, and G2/M phase of the cell cycle and represent mean ± s.d. of two sets of experiments in duplicate samples.

BCar is a microtubule inhibitor, therefore BCar treatment is expected to impede the mitotic (M) phases of the cell cycle. As shown in Fig. 4, treatment of BCar at ≥1 µM resulted in an accumulation of cells in the G2/M phase in both normal and cancer cells except for MDA-MB-175 VII, indicative of the G2/M cell cycle arrest. p53 active BC cells (*MDA-MB-175 VII* and *MDA-MB-361*) had a much larger fraction of the G1-phase cells after BCar treatment at ≥1 µM as compared to p53 inactive breast cancer cells (*MDA-MB-468*, *MDA-MB-231*, *HCC70*, *BT-549*), suggesting a stalled G1 phase of the cell cycle in the p53-wt cells. The important function of p53 is to activate the G1 cell cycle checkpoint response upon detecting the genotoxic insult to allow the repair of damage caused by the insult and protect the integrity of the cells [27]. However, if the genotoxic stress remains or the damage is irreversible, p53 triggers apoptosis to eliminate the involved cells [27]. A higher G1-phase population observed in the p53-wt cells treated with ≥1 µM BCar coincides with the significantly better survival of these cells compared to p53-mt BC cells (Figs. 2–4 and Table 1). Thus, the p53 role in the cell cycle checkpoint control appears to be a protective factor promoting repair and survival in the response to BCar.

### BCar induces mitotic catastrophe in p53-mutant breast cancer cells but not in p53-wild type breast cancer cells or normal HME cells

**Fig. 5:**
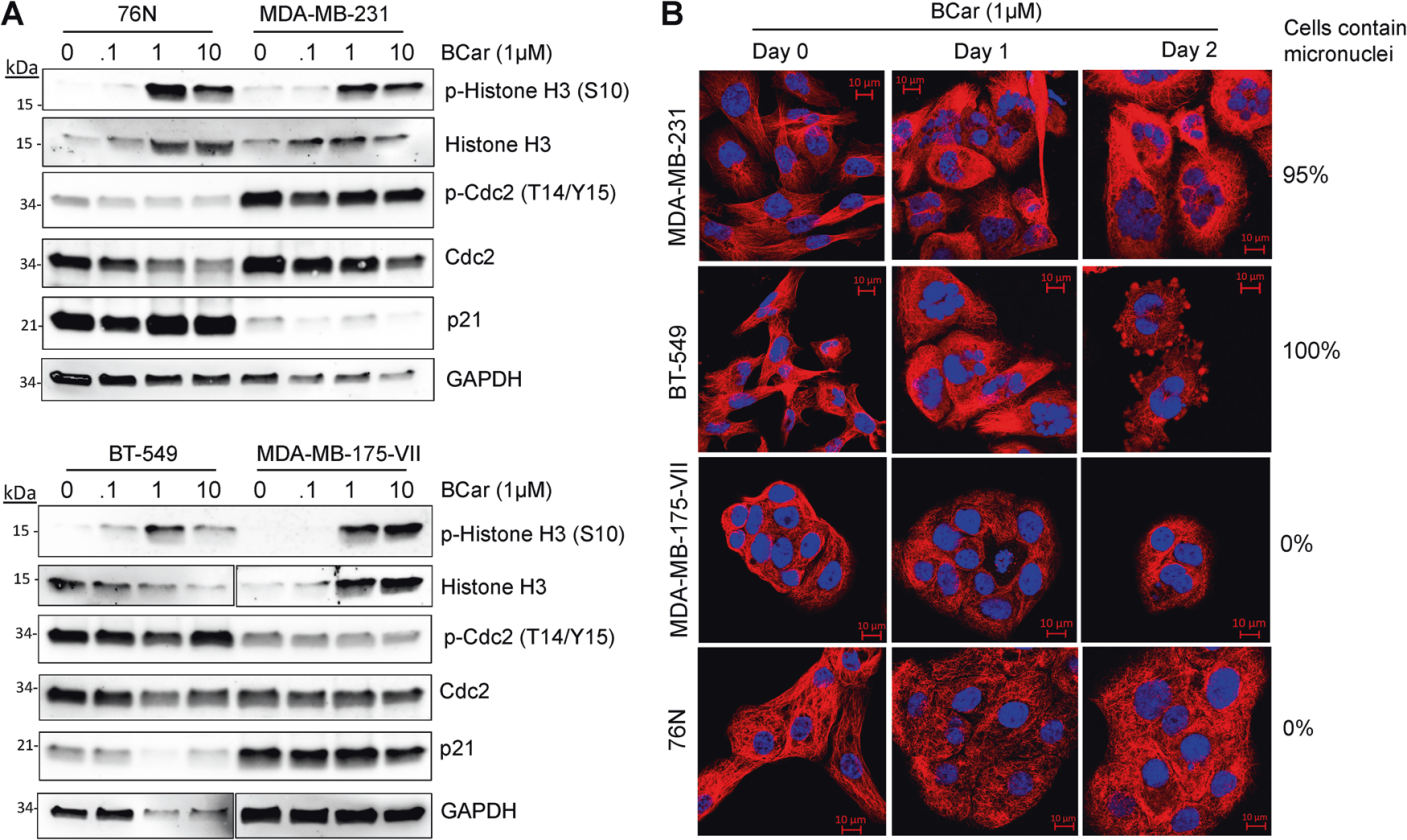
BCar-induced accumulation of multiple micronuclei, a hallmark of mitotic catastrophe, in p53-deficient breast cancer cells but not in p53-proficient normal HME or breast cancer cells. **A** Cells were incubated with increasing concentrations of BCar for 48 h and immunoblotted for the levels of Histone H3-S10 phosphorylation (a hallmark of mitotic cells), Cdc2-T14/Y15-phosphorylation (a hallmark of G2 checkpoint activation), and p21 (p53 target gene). GAPDH serves as a protein loading control. Note: Fig. 5A (lower panels): Histone H3 and GAPDH panels were from the same gels with different exposures. **B** Cells were incubated with 1 µM BCar for the times indicated, stained microtubules with an anti-α/β tubulin antibody, and nuclei with DAPI. The structure of microtubules and integrity of the nuclei were analyzed using a Zeiss-810 confocal laser-scanning microscope, as described in *Materials and Methods*. Scale bars, 10 μm.

Because both G2 and M phase cells contain 4N DNA content and cannot be distinguished from each other by measuring DNA content, we analyzed BCar-treated cells for phosphorylated-Cdc2-T14/Y15, the biomarker of G2 checkpoint activation, and phosphorylated-Histone H3-Ser10, the biomarker of mitotic cells. As shown in Fig. 5A, Western blotting revealed induction of Histone H3-S10 phosphorylation, indicative of mitotic checkpoint activation, in the cell lines treated with ≥1 µM BCar. In contrast, this treatment did not increase the phosphorylation of Cdc2-T14/Y15 in any of the cell lines (Fig. 5A), indicating the absence of the G2 checkpoint activation. These results confirm that the 4N-DNA content populations detected by the flow cytometry (Fig. 4) are cells in the M-phase. Furthermore, the effect of BCar on mitotic checkpoint response appears to be p53-independent (Figs. 4 and 5).

We also analyzed the level of p21, a p53 target that serves as a universal inhibitor of cyclin-dependent kinases and contributes to the G1 and G2 cell cycle checkpoint activation in response to genotoxic stress [27, 38]. As shown in Fig. 5A, the steady-state level of p21 is high in p53 active breast normal (76 N) and cancer (MDA-MB-175-VII) cells regardless of BCar’s concentration, and low in p53 inactive breast cancer (MDA-MB-231 and BT-549) cells. However, BCar treatment does not seem to alter the levels of p21 protein in the tested cell lines (Fig. 5A).

BCar binds to tubulins that assemble into spindle microtubules required for the mitotic phase to progress. We analyzed the structure of microtubules and the integrity of nuclei in BCar-treated cells. As shown in Fig. 5B, treatment with 1 µM BCar of p53 inactive MDA-MB-231 and BT-549 cells resulted in the induction of multi-micro-nucleated cells, indicative of mitotic catastrophe [39]. The same treatment of p53 active BC cells (MDA-MB-175-VII) or normal HME cells (76 N) (Fig. 5B) did not result in mitotic catastrophe. Furthermore, BCar-induced mitotic catastrophe in p53-mt breast cancer cells was correlated with greater induction of the M-phase cell population and significantly reduced clonogenic survival (Figs. 2, 4, and 5).

### Effects of BCar on apoptosis, autophagy, and senescence in normal HME and breast cancer cells

**Fig. 6:**
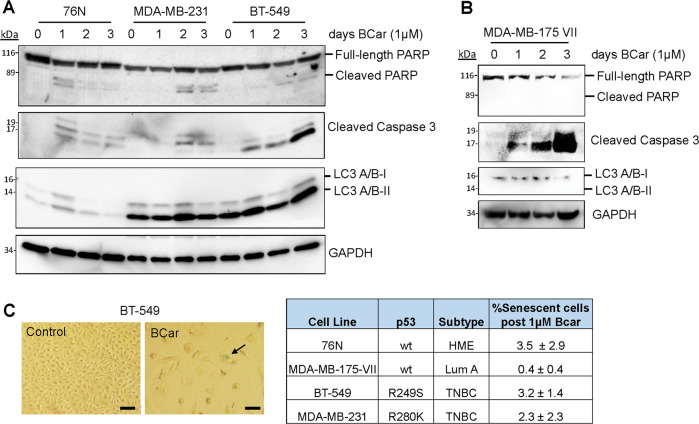
The effect of BCar on apoptosis, autophagy, and senescence in human normal mammary epithelial cells and breast cancer cells. **A**, **B** Log-phase growing cells were incubated with 1 µM BCar as indicated and analyzed for the integrity of PARP and caspase 3 cleavage (hallmarks of apoptosis), the levels of LC3A/B-I/II (a hallmark of autophagy) and GAPDH by Western blot analysis. **C** Cells were incubated with 1 µM BCar for 48 h, washed with plain medium, and then incubated for 1 week in regular growth medium. The resulting cells were analyzed for senescent cells with SA-β-gal assay. Blue-color-stained senescent cells were visualized by IMT-2 Olympus phase contrast microscope, quantified by ImageJ software and the percentage of senescent cells was analyzed by SigmaPlot software. The study was repeated twice in duplicates with similar results obtained. Arrow points at a senescent cell that is positive in β-galactosidase staining. Scale bars, 100 μm.

To comprehensively define mechanisms contributing to BCar-induced cytotoxicity, we analyzed the effects of BCar on other common cell death mechanisms induced by chemotherapy drugs, namely apoptosis, autophagy, and senescence [40, 41]. As shown in Fig. 6A, BCar treatment resulted in only a minor PARP and Caspase-3 cleavage in normal HME cells and p53-mt breast cancer cells. In contrast, BCar triggered a pronounced induction of PARP and Caspase-3 cleavage in p53-wt MDA-MB-175-VII BC cells. This response was time-dependent with a peak detected on day 3 post-treatment (Fig. 6B). It shall be noted that although Western blot did not detect cleaved PARP (89KD) in BCar-treated MDA-MB-175-VII cells, it revealed a time-dependent decrease in the levels of full-length PARP (116 KD) in these cells (Fig. 6B) indicating the loss of full-length PARP to cleavage. A similar case was reported in a study of MCF-7 BC cells [42]. Collectively, results in Fig. 6 suggest that p53 promotes the induction of apoptosis in response to the BCar-induced mitotic stress.

We next analyzed the induction of autophagy (also called type-II programmed cell death) in response to BCar by measuring the levels of LC3A/B-II, a biomarker of autophagy [43]. Autophagy is a self-degradative process that cleans out unnecessary cellular components or damaged cells. LC3A/B-II expression was detected at a moderate level in 76N cells and a much higher level in p53-mt BC cells (MDA-MB-231 and BT-549) (Fig. 6A). Overall, LC3A/B-II levels in these cells did not appear to change after exposure to BCar. However, in p53-wt MDA-MB-175-VII cells, LC3A/B-II expression was essentially undetectable either in the presence or absence of BCar (Fig. 6B). Accordingly, these results do not support autophagy as a mechanism contributing to the BCar-induced cytotoxicity.

We also assessed whether BCar induces stress-associated senescence. As shown in Fig. 6C, BCar-treatment resulted in a marginal increase in the number of senescent cells, as determined using the Senescence-Associated β-galactosidase activity (SA-β-gal) assay [44]. Thus, these results do not support the stress-associated senescence as a pathway contributing to the BCar-induced cytotoxicity.

### Knockdown of wild-type p53 by siRNA abrogates BCar-induced apoptosis in breast cancer cells and leads to mitotic catastrophe

**Fig. 7:**
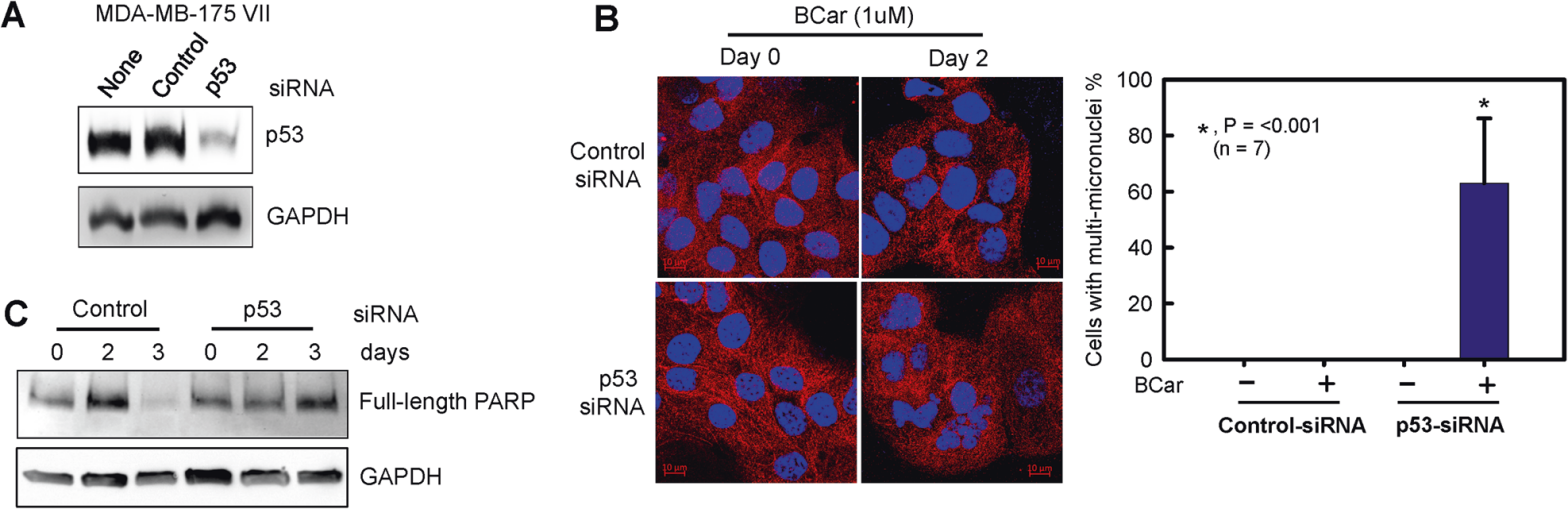
Knockdown of p53 and BCar treatment trigger the induction of mitotic catastrophe in MDA-MB-175-VII breast cancer cells, which is accompanied by the suppression of apoptosis induction. Cells were transfected with p53-siRNA for 48 h and then incubated with 1 µM BCar for 48 h. For the day 3 time point, cells were washed with plain medium after BCar treatment and incubated in growth medium for an additional 24 h before analysis. **A** Cell lysates were analyzed for p53 protein levels by Western blot. **B** Microtubule structures were visualized by immunostaining with an anti-α/β tubulin antibody. The nuclei were stained with DAPI and analyzed for their integrity by fluorescent confocal microscopy as described in Fig. 5B. Scale bar = 10 µm. Bar graph, the indicated cells were quantified for the percentage of cells containing multi-nuclei and the results are shown as mean ± s.d. of seven samples. **C**. Cell lysates were analyzed for the integrity of PARP protein and GAPDH by Western blot analysis.

To assess the p53 effect on the induction of mitotic catastrophe in BCar-treated breast cancer cells, wt-p53 was knocked down by siRNA in MDA-MB-175-VII cells. As shown in Fig. 7, the knockdown of p53 in MDA-MB-175-VII cells abrogated PARP cleavage following BCar treatment, indicating the absence of apoptotic events. Instead, the induction of multi-micronuclei phenotype was observed, indicating the induction of mitotic catastrophe. These results suggest the p53 role in the promotion of apoptosis induction and the inhibition of mitotic catastrophe, indicating that apoptosis and mitotic catastrophe may be mutually exclusive in responses to the BCar treatment.

While BCar activated the mitotic checkpoint in both p53-wt and p53-mt BC cells, it only induced mitotic catastrophe in p53-mt BC cells, which was also associated with greater cytotoxicity compared to p53-wt BC cells (Figs. 2–5 and Table 1). Corroborating these results, the knockdown of p53-wt in BC cells converted the cellular response to BCar from apoptosis to mitotic catastrophe (Fig. 7).

### BCar exhibits much less cytotoxicity in normal HME cells compared to clinical MTAs while maintaining comparable cytotoxicity in breast cancer cells

**Fig. 8:**
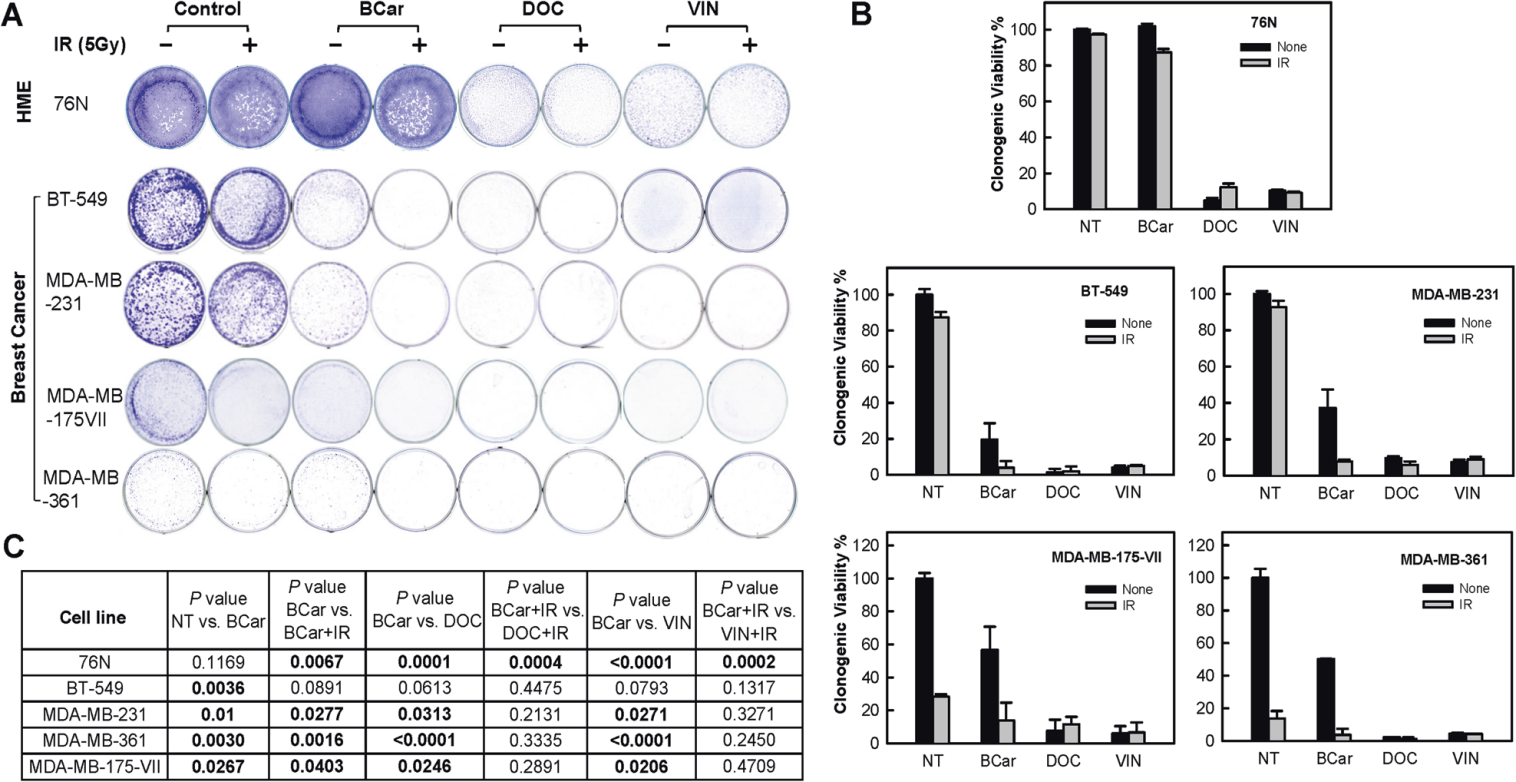
Comparison of BCar with clinical anti-mitotic drugs docetaxel (DOC) and vincristine (VIN) for the effect on the viability of human normal mammary epithelial cells and breast cancer cells. Cells were incubated with 0.5 µM BCar, DOC, or VIN for 24 h, exposed to 5 Gy radiation, and incubated for an additional 24 h. Cells were then washed and incubated for additional 7–14 days and analyzed for viability. **A** Representative samples from the cell viability assay. **B** Samples were quantified for cell survival using the ImageJ software. Results are expressed as mean ± s.d. of two sets of experiments in duplicate samples. **C** The EC_50_ of BCar among the breast normal and malignant cell lines were compared using an unpaired t-test. The *P* values showing significance (<0.05) from the comparisons are bolded.

To evaluate the clinical potential of BCar as an mBC therapeutic, we compared the effects of BCar to docetaxel (DOC) and vincristine (VIN), two frontline MTAs in the mBC treatment, in normal HME and BC cells. As shown in Fig. 8, incubation with 0.5 µM BCar produced no detectable cytotoxicity in 76 N cells at 14 days post-treatment. In contrast, the same dose of DOC and VIN treatment resulted in a 90–95% reduction in the viability of 76 N cells.

Since chemoradiation is a standard-of-care regimen for mBC [45], we evaluated the combined effect of MTAs and ionizing radiation (IR). Exposure to 5 Gy IR alone had a minimal effect on the survival of 76N. The combination treatment using 0.5 μM BCar and 5 Gy dose of IR decreased the viability of 76 N cells by ~10%. IR produced no additional toxicity in 76 N cells treated with DOC or VIN (Fig. 8).

We also compared the effects of BCar with DOC and VIN in BC cells expressing p53-mt and p53-wt. Treatment of p53-mt BT-549 and MDA-MB-231 cells with 0.5 µM BCar reduced their viability by ∼80% and ∼62%, respectively (Fig. 8). Furthermore, irradiation in the presence of BCar produced cytotoxicity far greater than BCar alone, resulting in ∼97% and ∼95% reduction in the viability of BT-549 and MDA-MB-231 cells, respectively (Fig. 8). In contrast, 5 Gy IR alone resulted in a minor reduction of the viability (5–10%) in BT-549 and MDA-MB-231 cells (Fig. 8). Treatment with 0.5 µM DOC or VIN alone reduced the viability of BT-549 and MDA-MB-231 cells by 95–97% and IR exposure had no additional measurable impact on the response. As shown in Fig. 8, treatment of p53-wt cells with 0.5 µM BCar alone reduced the viability of MDA-MB-175-VII and MDA-MB-361 cells by ∼42% and ∼50%, respectively. IR alone reduced the viability by ∼72% and ∼86% in MDA-MB-175-VII and MDA-MB-361 cells, respectively. A combined modality treatment decreased the viability of MDA-MB-175-VII and MDA-MB-361 cells by ∼86% and ~97%, respectively. However, the differences in the viability of cells treated with IR alone and cells treated with the combination of IR and BCar were not statistically significant (*P* = 0.3). Treatment with DOC or VIN at 0.5 µM reduced the viability of MDA-MB-175-VII and MDA-MB-361 cells by >90% and the addition of IR had no measurable impact (Fig. 8).

## Discussion

Microtubule-based anti-mitotic agents are commonly used for the therapy of mBC. However, MTAs are frequently associated with severe adverse events, and their widespread use results in the emergence of the drug-resistant disease, significantly limiting the usefulness of MTAs in the mBC treatment. Moreover, clinical MTAs act via similar mechanisms and so, when drug resistance develops, it often involves all MTAs [6, 7, 9, 10, 12]. In patients who develop MTA resistance, the subsequent MTA treatments are less effective and may produce additional side effects. Thus, there is a critical need to develop novel MTAs that are safer and act through different mechanisms thereby avoiding the shortcoming of clinically used MTAs.

Two types of MTAs currently used as chemotherapeutics include the microtubule stabilizers binding to the taxane site and microtubule destabilizers binding to the vinca site [4]. While both types interrupt the microtubule function and thereby kill cancer cells, the precise mechanisms underlying MTA-induced cell death remain uncertain [4]. Apoptosis is commonly used to assess MTA cytotoxicity. However, many studies report the absence of any direct correlations between the extent of the MTA-induced mitotic arrest and the level of apoptosis, suggesting that the alternative cell death mechanisms may be triggered by MTAs [46–48]. Taxanes are most often used in mechanistic studies aimed to determine cell death pathways. For example, it is reported that, in BC cells, high concentrations of Taxol primarily cause necrosis, whereas low concentrations mainly result in apoptosis [49, 50]. Other mechanisms were also described in taxane-treated cells and include pyroptosis, mitotic catastrophe, and autophagy [51]. Although the exact determinants of the taxane death response remain largely undefined, the cell type and p53 mutational status are among the factors of concern. In the current study, we assessed the effects of BCar on clonogenic cell survival in the context of apoptosis, senescence, mitotic catastrophe, and autophagy using a panel of BC cells, with/without p53 function, and in normal breast cells. The results indicate that the BCar-induced BC cell death involves two distinct mechanisms, the p53-dependent apoptosis in p53-wt BC cells and the p53-independent mitotic catastrophe in p53-mt BC cells.

**Fig. 9:**
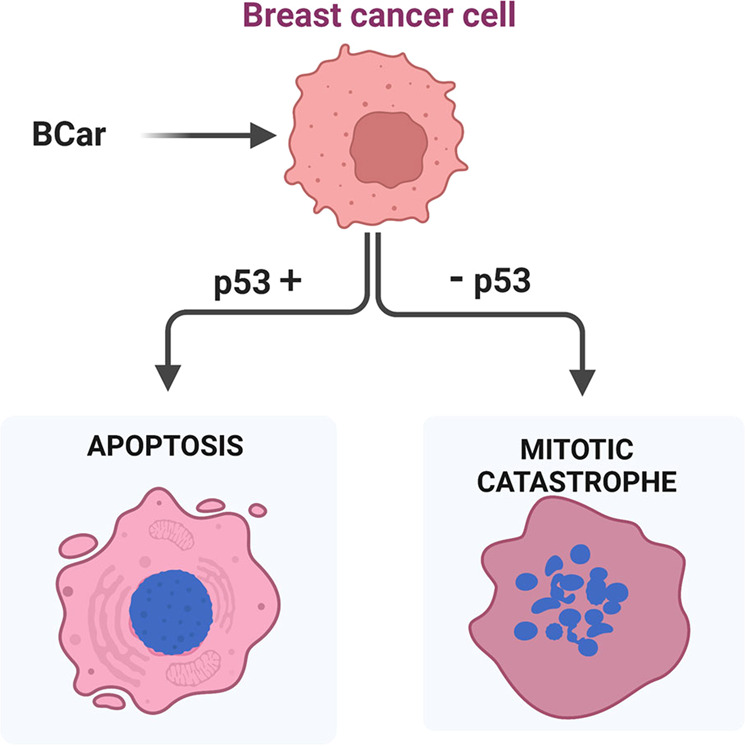
A conceptual model of the mechanisms of BCar cytotoxicity. BCar can trigger p53-dependent apoptosis in p53 proficient (p53+) breast cancer cells, while it induces mitotic catastrophe in breast cancer cells in p53 deficient (p53−) cells.

Mutant p53 is detected in >80% of metastatic breast cancer and is considered a driver of the progression of breast cancer into metastatic disease [28–30]. In response to genotoxic stress, p53 rapidly activates the G1 checkpoint response by inducing p21^Cip1^, which in turn directly inhibits Cdk4/6 kinases, thus arresting cells at the G1/S border of the cell cycle to repair the damage [27]. However, if the damage is persistent, apoptosis is triggered to remove the affected cells and protect the genomic integrity [27]. The genotoxic stress-induced G2/M cell cycle checkpoint response is mainly a p53-independent event and it requires the induction of ATM/ATR signaling pathways [52, 53]. Consistent with previous studies, our results revealed two distinct death mechanisms activated by BCar, the p53-dependent apoptosis and the p53-independent mitotic catastrophe (Fig. 9). The p53-independent mitotic catastrophe occurs within 24 h after the BCar-treatment. The p53-dependent apoptosis is a delayed event showing a maximum induction at ~72 h after BCar-treatment. These conclusions were validated using siRNA knockdown of p53-wt in BC cells. The response to BCar reversed from the expected apoptosis to the rapid mitotic catastrophe. It is therefore important to point out that since p53-mt BC cells are more sensitive to BCar treatment than p53-wt breast cancer cells, BCar is anticipated to be effective in treating mBC expressing p53-mt.

Ideal anticancer drugs are those that can induce cancer cell death while sparing normal tissues. In addition, the ability to overcome drug resistance is advantageous. In this respect, BCar-based drugs are good clinical candidates. Moreover, they can be administered orally and have over four decades of clinically proven safety records as FDA-approved anthelmintics [13]. The low cytotoxicity of BCar in normal HME cells further underscores its potential as a safe therapeutic. While BCar cytotoxicity in HME cells is far lower than DOC and VIN, this agent maintains a comparable killing activity in BC cells. BCar binds to the colchicine site that is remote from the binding domains of established chemotherapeutic MTAs [13]. For that reason, BCar-based agents can be expected to be efficacious as the second-line treatment in the MTA-resistant mBCs. It is worth noting that BCar does not cause mitotic catastrophe in HME cells and p53-wt BC cells. However, BCar treatment still results in 7–10 folds higher toxicity in p53-wt BC cells than in normal HME cells. The molecular basis for this difference is unclear but suggests the presence of p53-independent mechanism(s) that promote the death of p53-wt BC cells while sparing normal HME cells from the cytotoxic effect of BCar. Future studies are required to elucidate the cellular mechanisms underlying these differences.

In summary, this study evaluated BCar, a microtubule destabilizer, for its cytotoxic effects in human normal HME cells and BC cells and uncovered the differences in the operational death mechanisms associated with the p53 status. Our data indicate that BCar has one order of magnitude greater cytotoxicity against BC cells than normal HME. Additionally, p53-inactive (p53-mt) BC cells are much more sensitive to BCar treatment than p53-active (p53-wt) BC cells. Two distinct cell death mechanisms appear to be triggered by BCar, the p53-dependent apoptosis, and the p53-independent mitotic catastrophe. Lastly, under identical conditions, BCar shows far less cytotoxicity in normal HME cells than clinical DOC and VIN while maintaining a similar killing activity in cancer cells. The large differences between the effective and toxic concentrations of BCar indicate that a wider therapeutic window may be attained compared to DOC and VIN. Overall, our report provides strong evidence supporting BCar as a new line of MTAs for mBC.

## Materials and methods

### Cell lines, reagents, and treatment

Human breast cancer cell lines MDA-MB-231, MDA-MB-468, MDA-MB-361, MDA-MB-175-VII, HCC70, and BT-549 were obtained from American Type Culture Collection (ATCC, Manassas, VA) and maintained in Dulbecco’s Modified Eagle’s Medium (DMEM) containing 10% fetal bovine serum (FBS). MCF10A, a nontumorigenic human mammary epithelial cell line spontaneously immortalized from fibrocystic breast tissue, was obtained from ATCC. 76N, a cell line derived from human primary mammary epithelial cells immortalized by human telomerase (hTERT) [36], is a kind gift from Dr. Vimla Band (University of Nebraska Medical Center). MCF10A and 76N were maintained in Mammary Epithelial Growth Medium (MEBM) supplemented with Bullet Kit from Lonza Bioscience (Morrisville, NC) and 1% FBS, respectively. Methyl *N*-(6-benoyl-1*H*-benzimidazol-2-yl)carbamate (BCar), was purchased from Grainger (Lake Forest, IL) and analyzed using previously described HPLC methods [54] to ensure the chemical purity of >96%. BCar was used in a dimethyl sulfoxide (DMSO) solution. Docetaxel and Vincristine were purchased from Selleck Chemicals (Houston, TX) and dissolved in DMSO. For drug treatment, cells were incubated for 48 h in media containing specified drug concentrations. For experiments involving both drug treatment and IR exposure, exponentially growing cells were incubated with drug-contained media for 24 h, exposed to IR, and followed by additional 24-h incubation in the presence of the drug at 37 °C. Control cells were grown in a medium containing DMSO at a final concentration of ≤0.1%.

### Cell viability assay

Cell viability assay was performed as described previously [42]. Log-phase growing cells were incubated in the media containing BCar at the indicated concentrations for 48 h, washed with plain medium, and incubated in growth medium for 7–14 days. Cell viability (surviving cells or formed colonies) was visualized by staining with crystal violet (Sigma-Aldrich, St. Louis, MO), scanned using an EPSON Perfection 4490PHOTO scanner, quantified with the Fiji-ImageJ analytical program (NIH, Bethesda, MD) and analyzed by SigmaPlot 11.2 software.

### Trypan blue exclusion test of cell viability

Cells were plated at approximately 25% confluency in 6-well plates, treated with BCar at the indicated concentrations for 0–3 days, and then harvested by trypsinization. Equal volumes of the cell suspension and 0.4% solution of trypan blue in PBS were mixed to stain dead cells. Cell viability was determined using a Cellometer Auto 2000 (Nexcelom, Lawrence, MA) as instructed by the manufacturer.

### Cell cycle analysis

BCar-treated and untreated control cells were harvested, fixed following the standard protocol, and stained with Telford reagent. DNA content was analyzed by fluorescence-activated cell sorting using a FACSClibur instrument (Beckon Dickinson, Mansfield, MA) as described previously [55]. Each analysis was performed using 20,000 cells.

### Antibodies and Western Blot analysis

Antibodies were purchased from Cell Signaling Technologies (Danvers, MA) unless otherwise indicated. These include mouse IgG against Caspase 8 (1C12), Histone H3 (1G1) (Santa Cruz Biotechnology, Santa Cruz, CA), Cdc-2 (17) (Santa Cruz Biotechnology), and GAPDH (Santa Cruz Biotechnology); rabbit IgG against p21, PARP, Cleaved Caspase 3 (Asp175) (5A1E), LC3A/B (D3U4C), α/β tubulin, p-Cdc-2 (T14/Y15) (Santa Cruz Biotechnology), p53 (FL-393) (Santa Cruz Biotechnology), and p-Histone H3 (Ser10) (Upstate Cell Signaling Solutions, Lake Placid, NY). Secondary antibodies used for immunofluorescence include donkey anti-rabbit IgG for Alexa Fluor 594 (Invitrogen, Waltham, MA). Nuclei were visualized by DAPI (Sigma-Aldrich, St. Louis, MO) staining at 1 µg/ml for 5 min.

Western Blot analyses were carried out as previously described [55]. Western blots were visualized by chemiluminescence using Bio-Rad ChemiDoc™ Imaging System (Hercules, CA) and specific protein signals were quantified using the Fiji-ImageJ analytical software (NIH, Bethesda, MD).

### Short interfering RNAs and transfection

Short interfering RNA (siRNA) duplexes were obtained from Dharmacon Research (Lafayette, CO). Nontargeting control siRNA contained at least four mismatches to any human, mouse, or rat gene, as determined by the manufacturer. The sequence for control siRNA is 5′-UAAGGCUAUGAAGAGAUAC-3′. SMARTpool siRNA targeting TP53 consists of four siRNA targeting multiple sites on TP53. siRNA sequences are 5′-GAAAUUUGCGUGUGGAGUA-3′, 5′-GUGCAGCUGUGGGUUGAUU-3′, 5′-GCAGUCAGAUCCUAGCGUC-3′, 5′- GGAGAAUAUUUCACCCUUC-3′. Cells were transfected with siRNA at 100 nM using DharmaFECT1 siRNA transfection reagent (Dharmacon Research) according to the manufacturer’s instruction. In experiments involving both siRNA transfection and BCar treatment, cells were incubated for 48 h after transfection prior to BCar treatment.

### Immunofluorescence and microscopy

Immunofluorescence and microscopy were performed as described previously [56]. Briefly, cells grown on coverslips (#1-thickness) were treated with 1 µM BCar for the time indicated. BCar-treated and control DMSO-treated cells were fixed in 4% paraformaldehyde (PFA) for 20 min and permeabilized with 0.25% Triton X-100 for 10 min. Cell samples were then blocked for 30 min with 10% horse serum, 1% bovine serum albumin (BSA), and 0.5% Tween-20 in Tris-buffered saline (TBS) at ambient temperature. To analyze the microtubule structure, cells were incubated overnight at 4 °C in anti-α/β tubulin rabbit IgG (#2148, Cell Signaling) at 1:50 dilution in TBS containing 1% BSA/0.05% Tween-20. After washing three times with 1% BSA in TBS, cells were incubated with Alexa Fluor 594 anti-rabbit IgG (1:200 dilution, Cat. #A-21207) for 1 h in the dark. DAPI staining occurred at 1 µg/ml for 5 min also in dark. Images of immune-stained cells were taken using a Zeiss LMS800 Airyscan confocal laser-scanning microscope (Hitech Instruments, Pennsburg, PA). Phase-contrast cell images were visualized using an IMT-2 Olympus phase contrast microscope.

### SA-β-gal assay

The SA-β-gal assay was performed as described previously [44]. Briefly, cells were fixed in 2% formaldehyde/0.2% glutaraldehyde for 5 min, washed with PBS, and stained with 1 mg of 5-bromo4-chloro-3-indolyl β-D-galactoside (X-Gal) per ml in assay buffer (40 mM citric acid/sodium phosphate, pH 6.0; 5 mM potassium ferrocyanide; 5 mM potassium ferricyanide; 150 mM NaCl; 2 mM MgCl_2_) for overnight at 37 ^o^C.

### Statistical analysis

Student’s *t* test and one-way ANOVA methods were used for the comparison of experimental groups using SigmaPlot 11.2 software (Palo Alto, CA). The half-maximal effective concentrations (EC_50_) of BCar for the inhibition of cell survival were calculated with the nonlinear regression functions of GraphPad Prism 9 (San Diego, CA). EC_50_ comparison statistics were performed by unpaired *t* test. *P* values ≤0.05 were considered significant.

## Supplementary information

Original Western blots for Figure 5

Original Western blots for Figure 6

Original Western blots for Figure 7

## Data Availability

All data generated or analyzed during this study are included in the published article.
