# Supplementary material for: Benzimidazole carbamate induces cytotoxicity in breast cancer cells via two distinct cell death mechanisms
Source: Cell Death Discov. 2023 May 13;9:162. doi: 10.1038/s41420-023-01454-6 (PMC10183037; doi:10.1038/s41420-023-01454-6)
Supplement: Supplementary file 1 — Original Western blots for Figure 5 [file 41420_2023_1454_MOESM1_ESM.pdf]

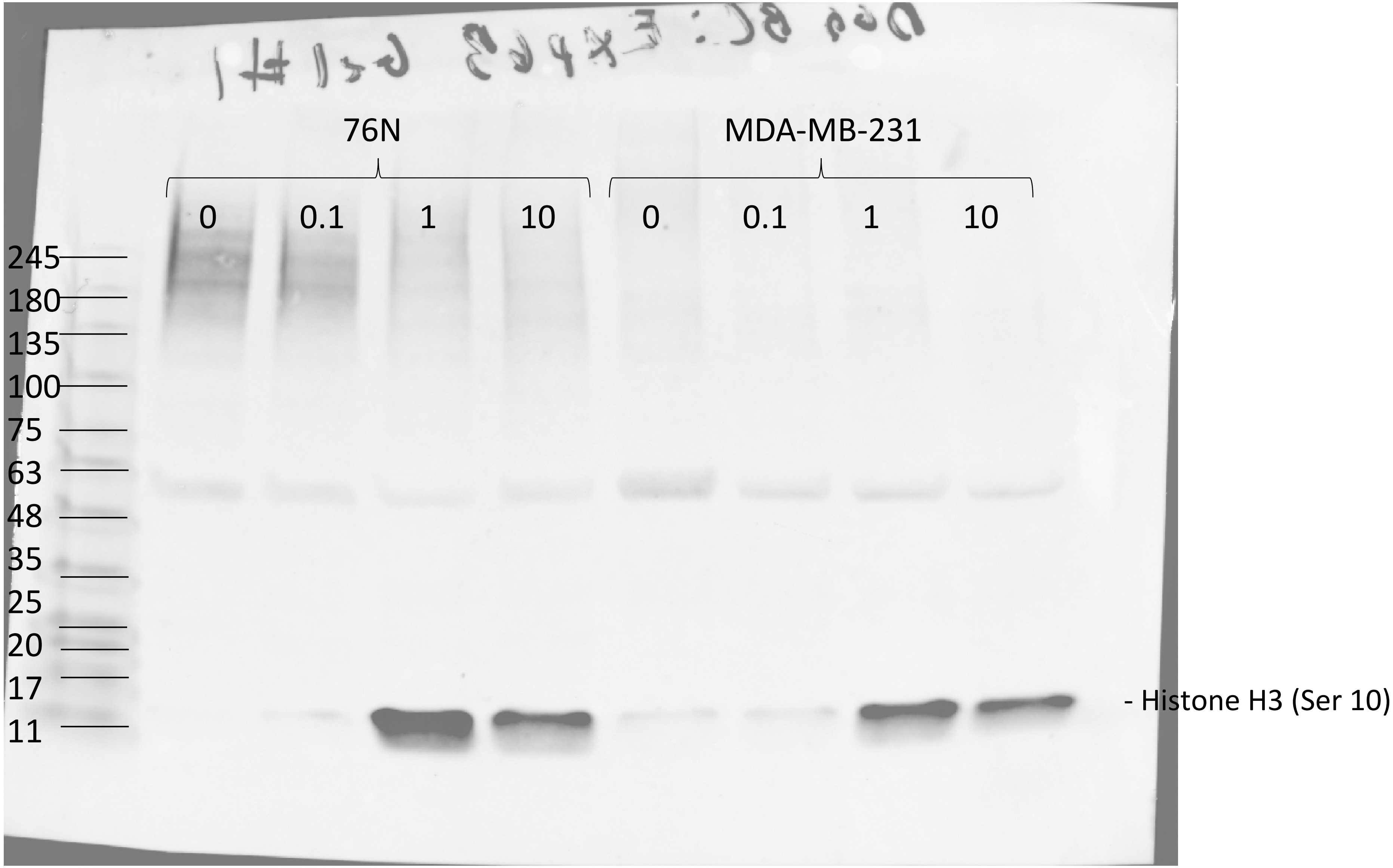

Fig. 5A, Top panel: Histone H3 (Ser 10)
1st antibody: Rb: Upstate 06-587 (1:500 TBS)
2nd antibody Ms anti-Rb: 711-035-152 (1:5000 5% milk)

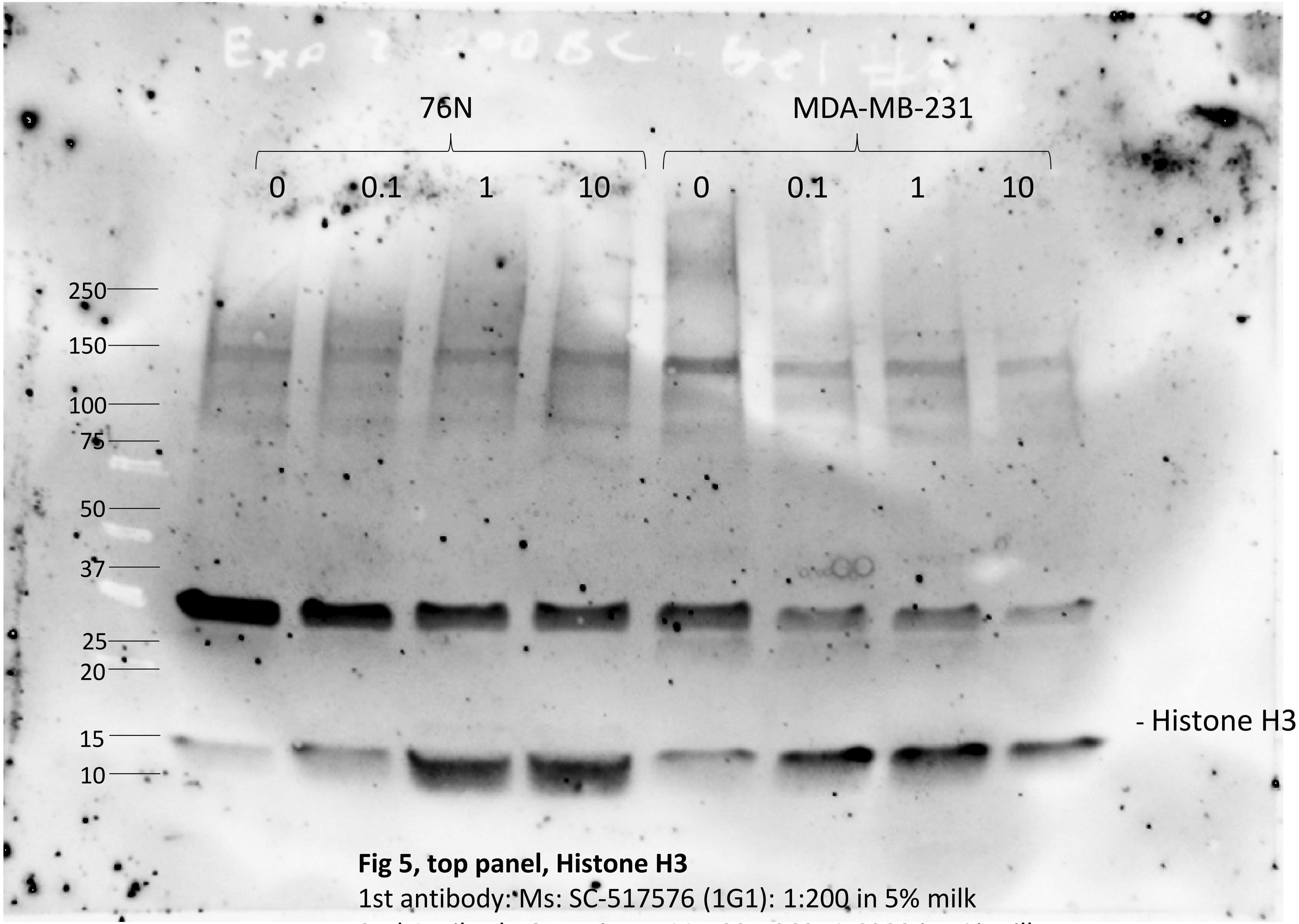

Fig 5, top panel, Histone H3

1st antibody: Ms: SC-517576 (1G1): 1:200 in 5% milk

2nd Antibody Gt anti-Ms: 115-035-062: 1:6000 in 5% milk

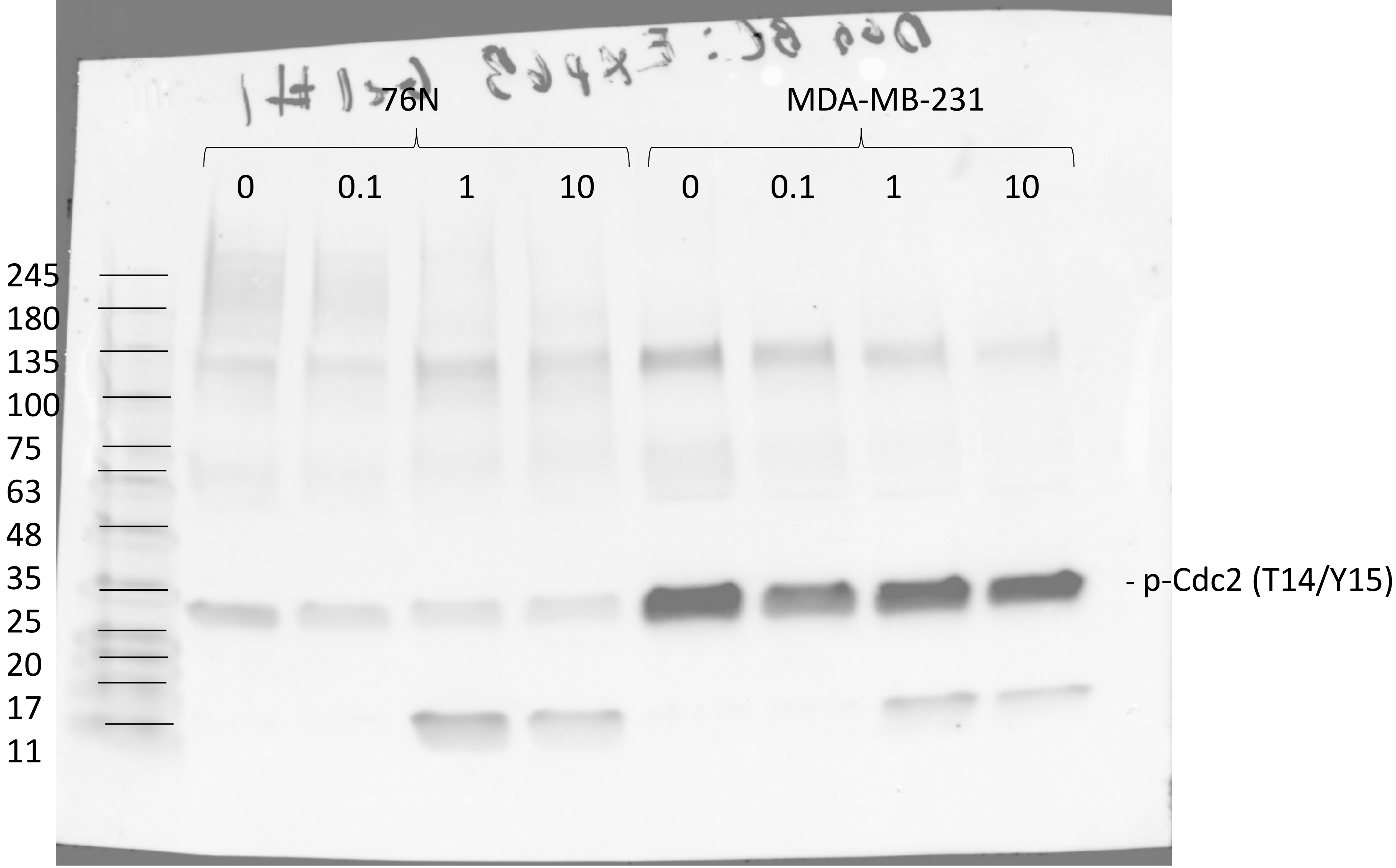

Fig 5A, top panel, p-Cdc2 (T14/Y15)

1st antibody: Rb; Santa Cruz, Cat# SC-12340-R, 1:1000 in TBS 2nd antibody: Ms anti-Rb; 711-035-152 (Jackson) 1:6000 in 5% milk

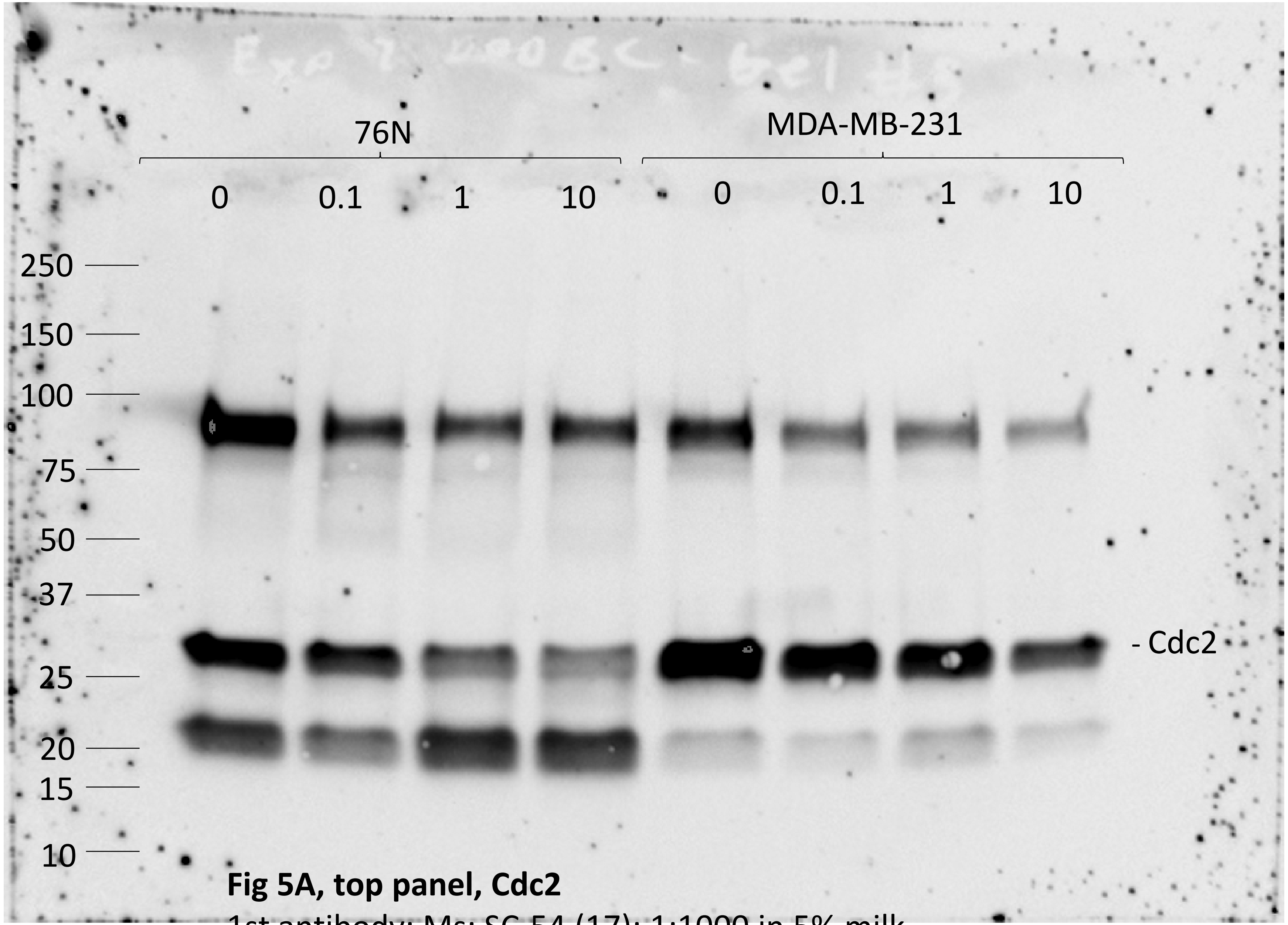

Fig 5A, top panel, Cdc2

1st antibody: Ms: SC-54 (17): 1:1000 in 5% milk

2ne antibody: Gt anti-Ms: 115-035-062: 1:5000 in 5% milk

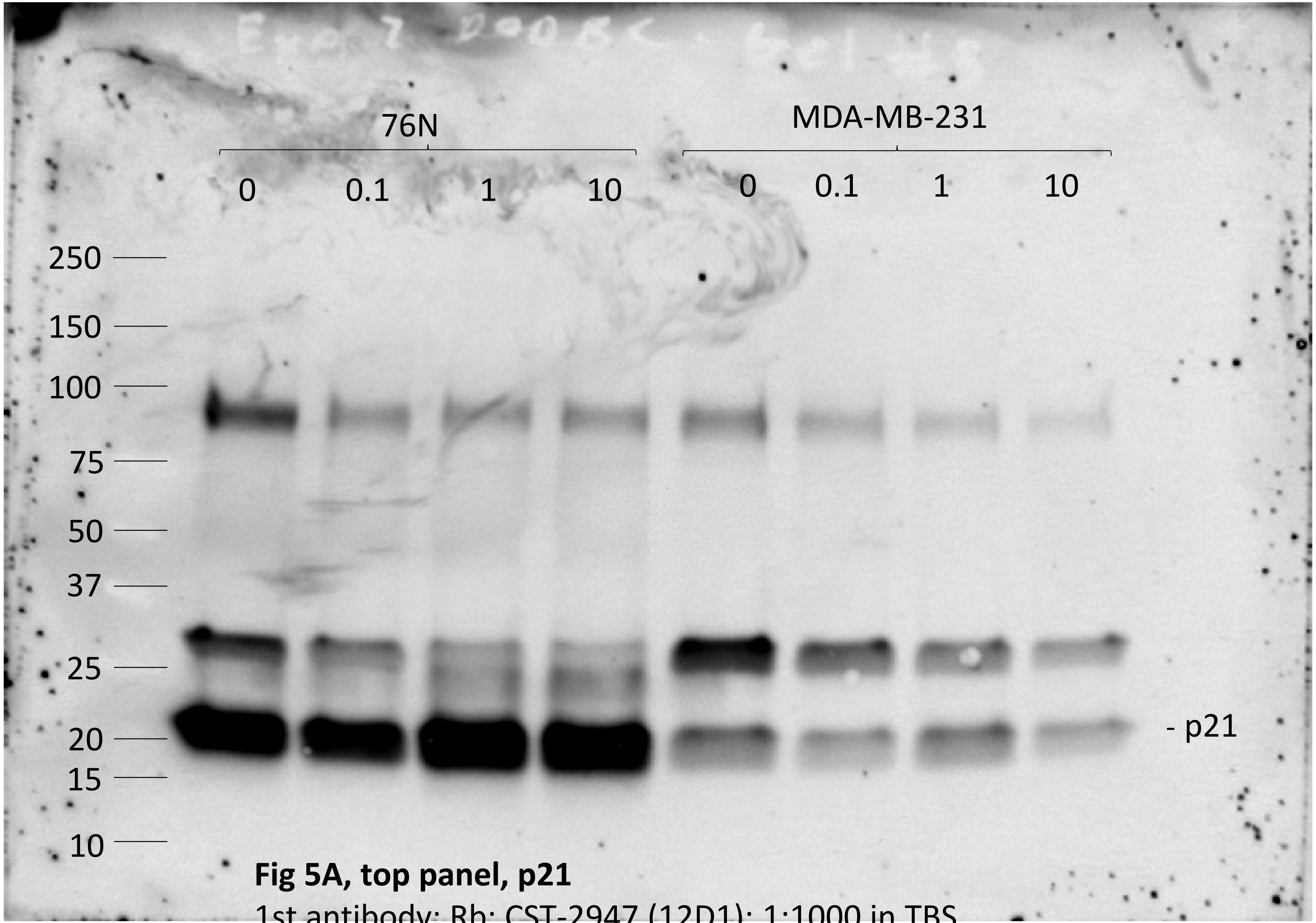

Fig 5A, top panel, p21
1st antibody: Rb: CST-2947 (12D1): 1:1000 in TBS
2nd antibody: Ms anti-Rb: 711-035-152: 1:5000 in 5% milk

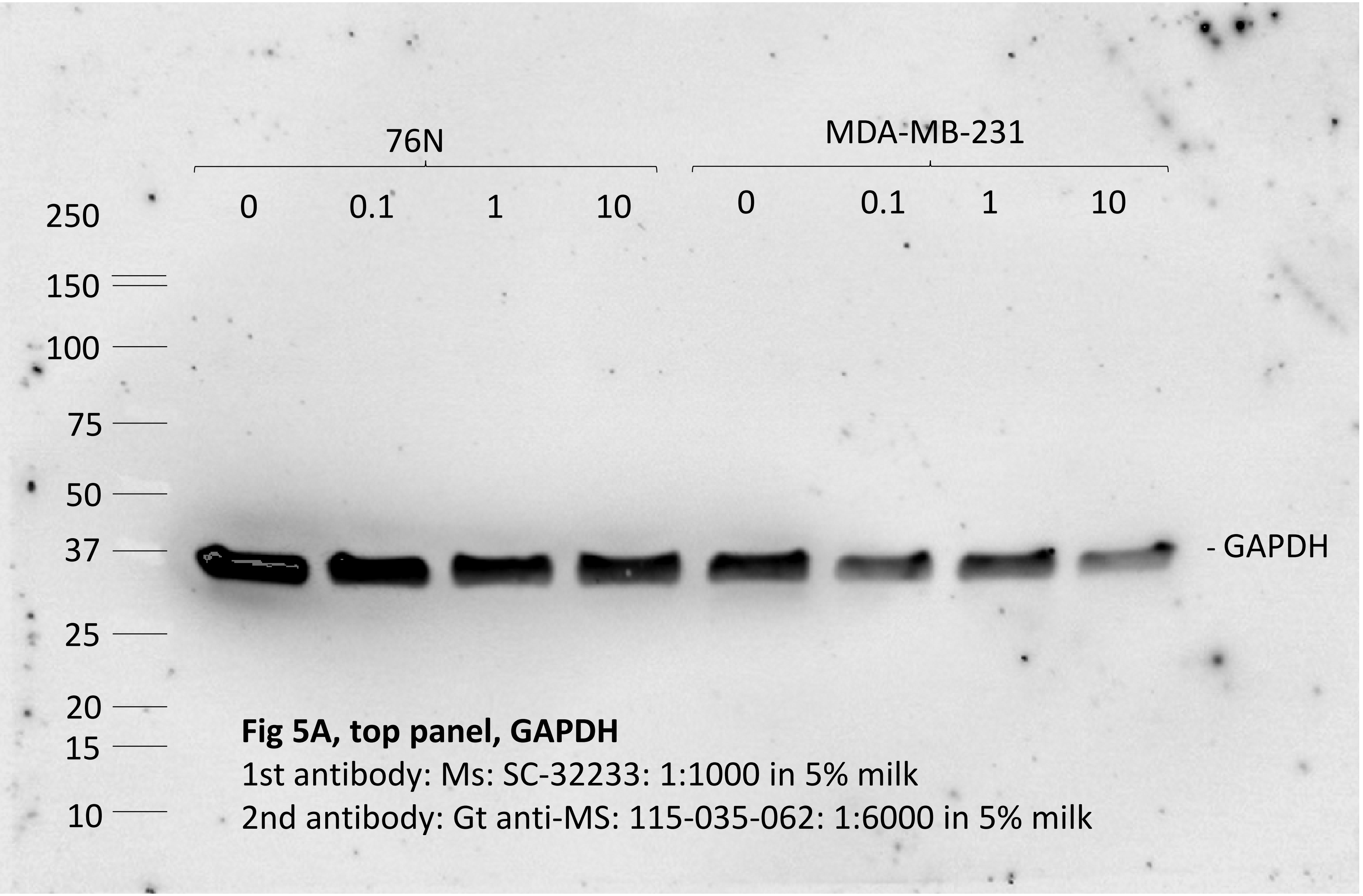

Fig 5A, top panel, GAPDH
1st antibody: Ms: SC-32233: 1:1000 in 5% milk
2nd antibody: Gt anti-MS: 115-035-062: 1:6000 in 5% milk

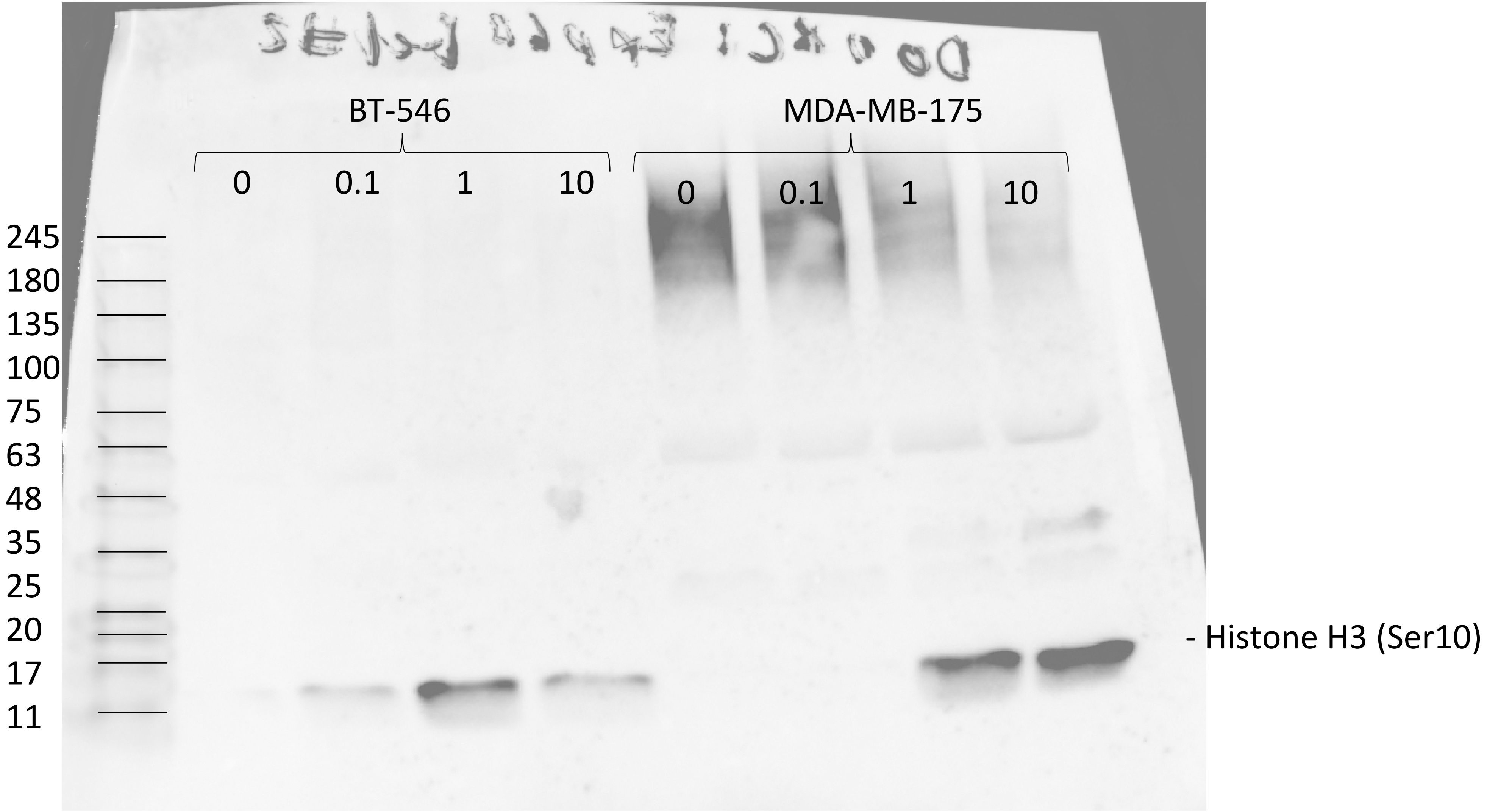

Fig 5A, bottom panel, Histone H3 (Ser 10)
1st antibody: Rb: Upstate 06-587 (1:500 TBS)
2nd antibody: Ms anti-Rb: 711-035-152 (1:5000 5% milk)

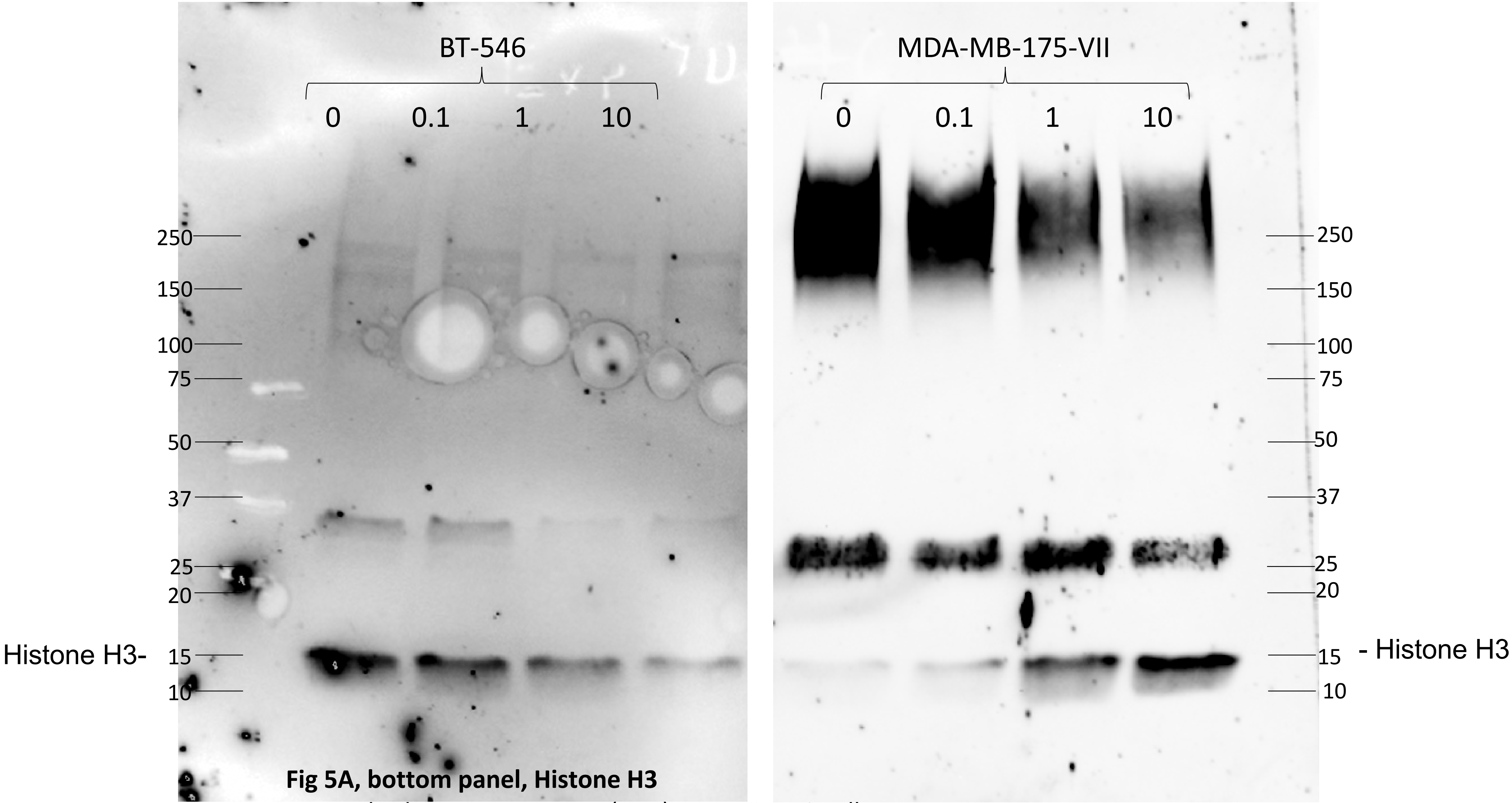

Fig 5A, bottom panel, Histone H3

1st antibody: Ms: SC-517576 (1G1): 1:200 in 5% milk

2nd antibody: Gt anti-Ms: 115-035-062: 1:6000 in 5% milk

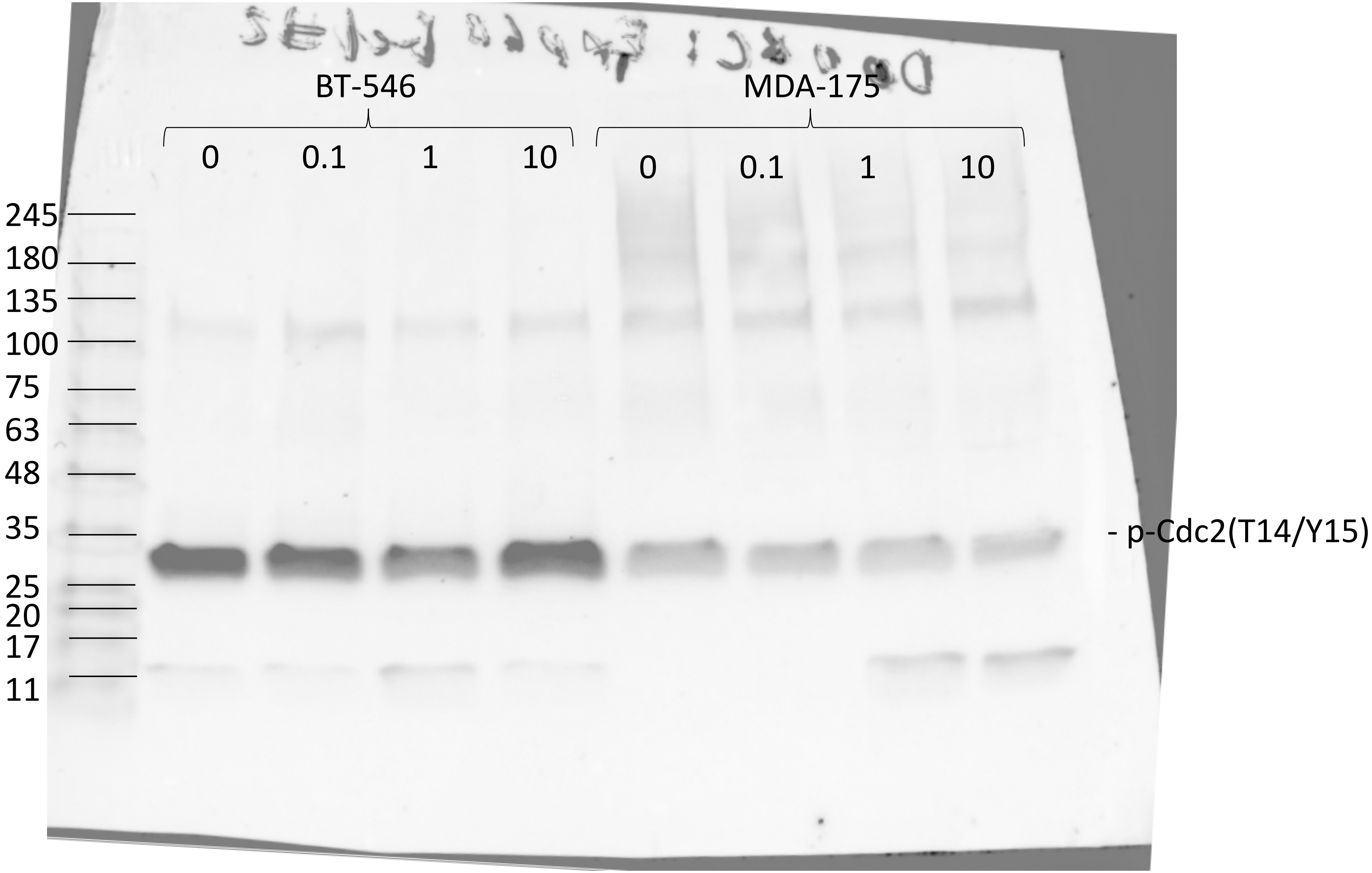

Fig 5A, bottom panel, p-Cdc2 (T14/Y15)

1st antibody: Rb; Santa Cruz, Cat# SC-12340-R, 1:1000 in TBS

2nd antibody: Ms anti-Rb; 711-035-152 (Jackson) 1:6000 in 5% milk

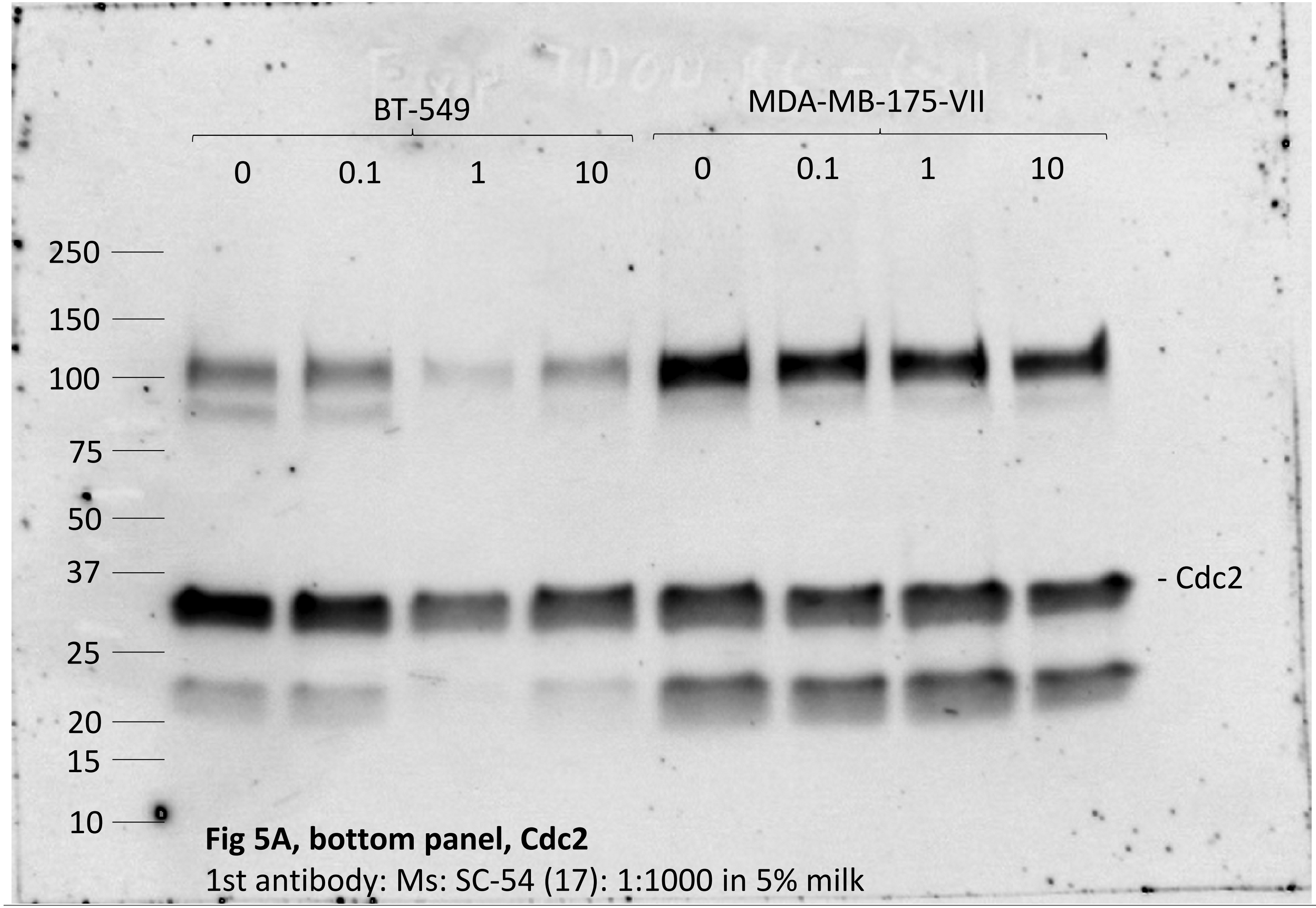

Fig 5A, bottom panel, Cdc2

1st antibody: Ms: SC-54 (17): 1:1000 in 5% milk

2nd antibody: Gt anti-Ms: 115-035-062: 1:5000 in 5% milk

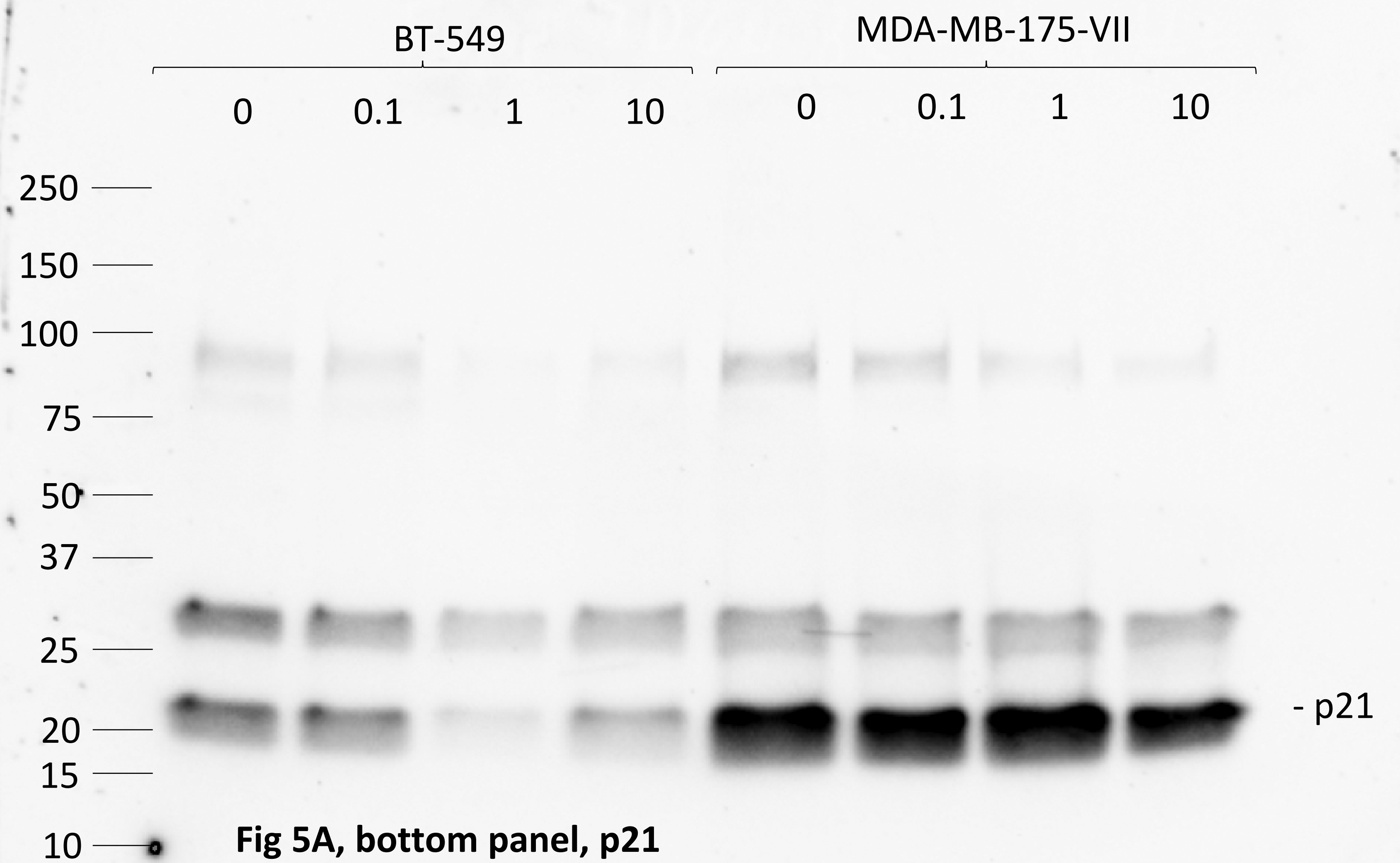

Fig 5A, bottom panel, p21

1st antibody: Rb: CST-2947 (12D1): 1:1000 in TBS

2nd antibody: Ms anti-Rb: 711-035-152: 1:5000 in 5% milk

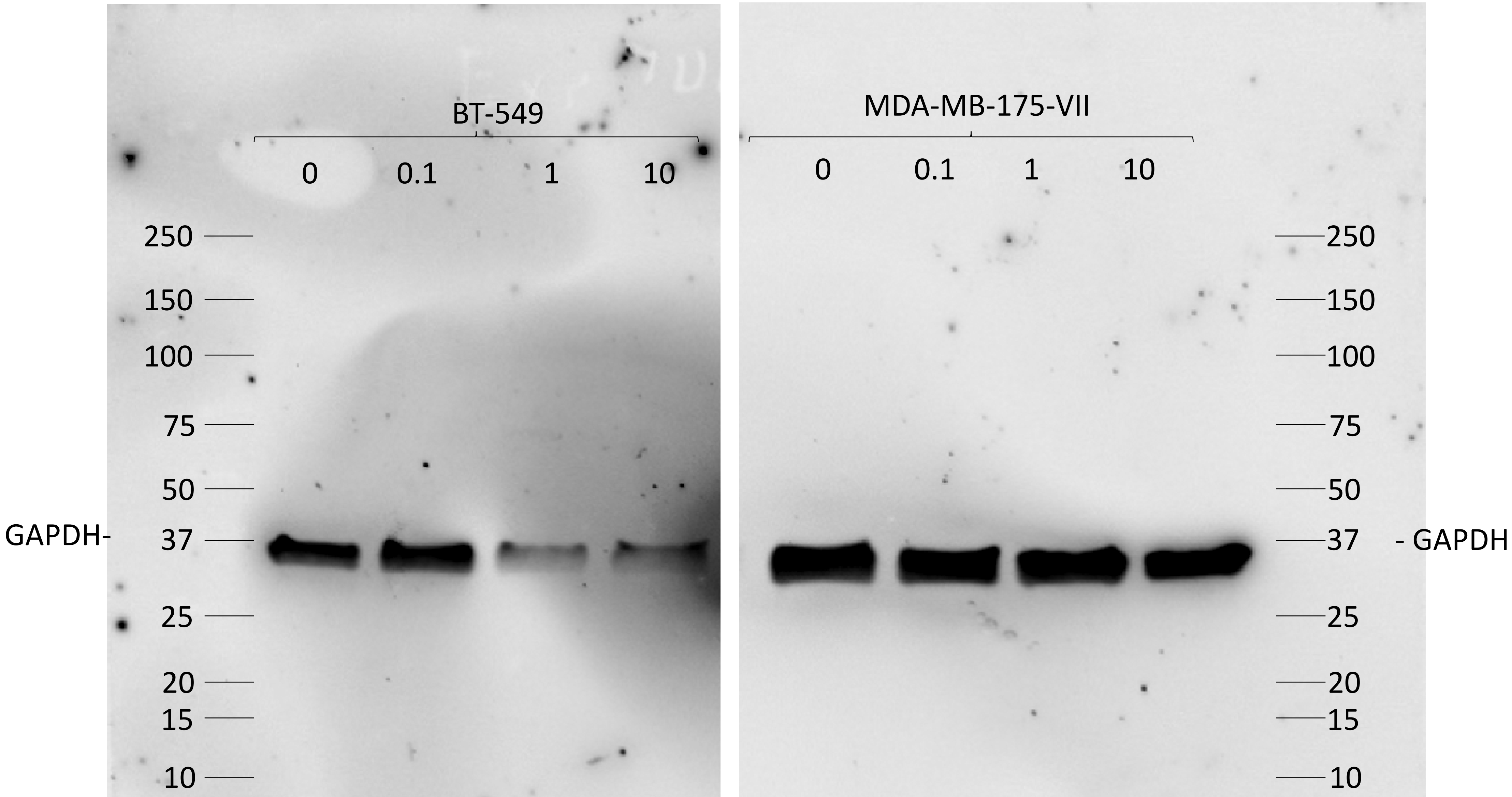

Fig 5A, bottom panel, GAPDH

1st antibody: Ms: SC-32233: 1:1000 in 5% milk

2nd antibody: Gt anti-MS: 115-035-062: 1:6000 in 5% milk
