# Supplementary material for: Benzimidazole carbamate induces cytotoxicity in breast cancer cells via two distinct cell death mechanisms
Source: Cell Death Discov. 2023 May 13;9:162. doi: 10.1038/s41420-023-01454-6 (PMC10183037; doi:10.1038/s41420-023-01454-6)
Supplement: Supplementary file 2 — Original Western blots for Figure 6 [file 41420_2023_1454_MOESM2_ESM.pdf]

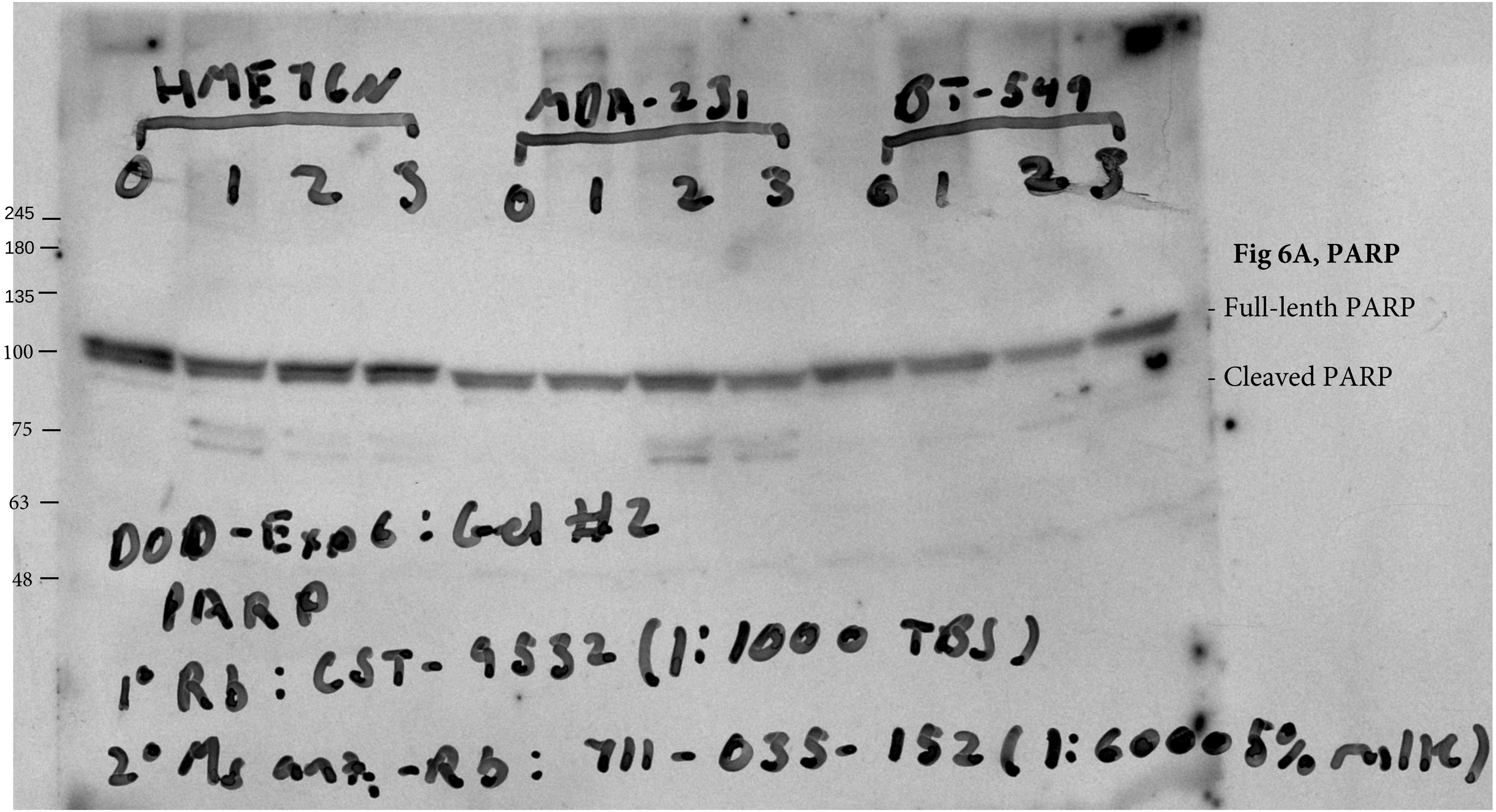

Fig 6A, PARP
- Full-lenth PARP
- Cleaved PARP
245
180
135
100
75
63
48
HMEC
MDA-231
BT-549
0 1 2 3
0 1 2 3
0 1 2 3
PARP
1° Rb : CST-9532 (1:1000 TBS)

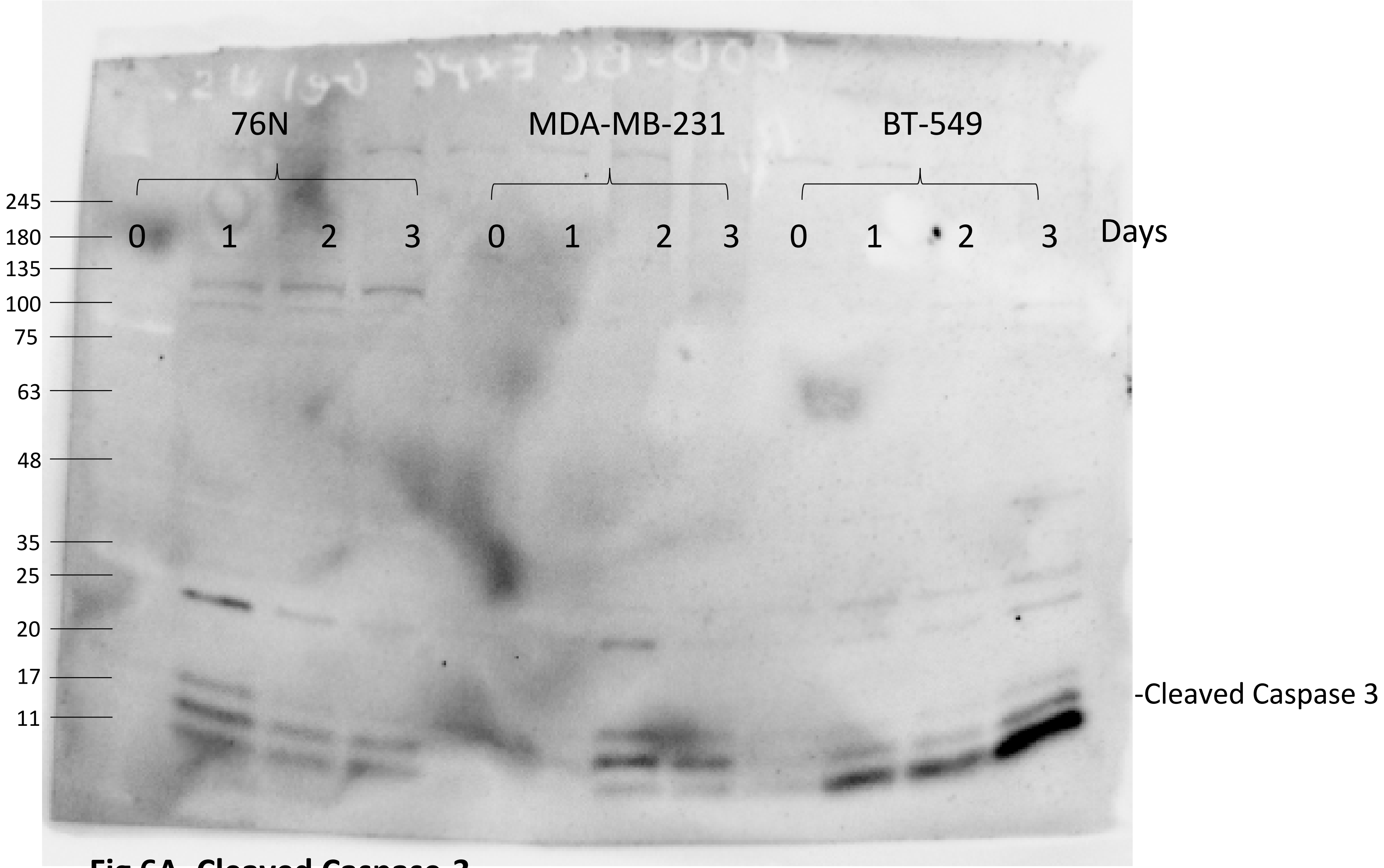

Fig 6A, Cleaved Caspase-3

1st antibody: Rb: CST-9664 (1:1000 in TBS)

2nd antibody: Ms anti-Rb: 711-035-152 (1:6000 in 5% milk)

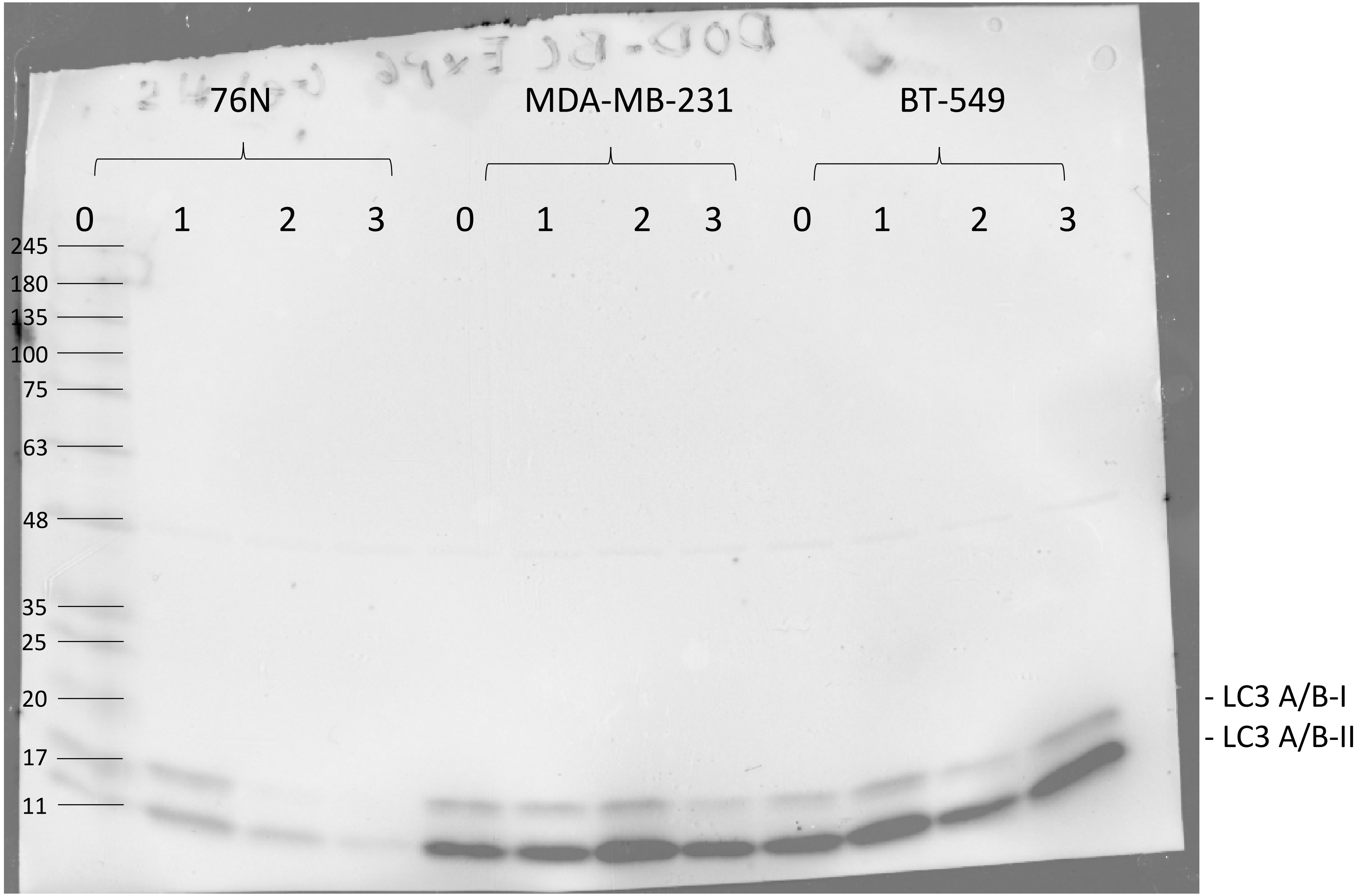

Fig 6A, LC3A/B-I/II

1st antibody: Rb; CST-12741; 1:1000 TBS

2nd antibody: Ms anti-Rb; 711-035-152: 1:6000 5% milk

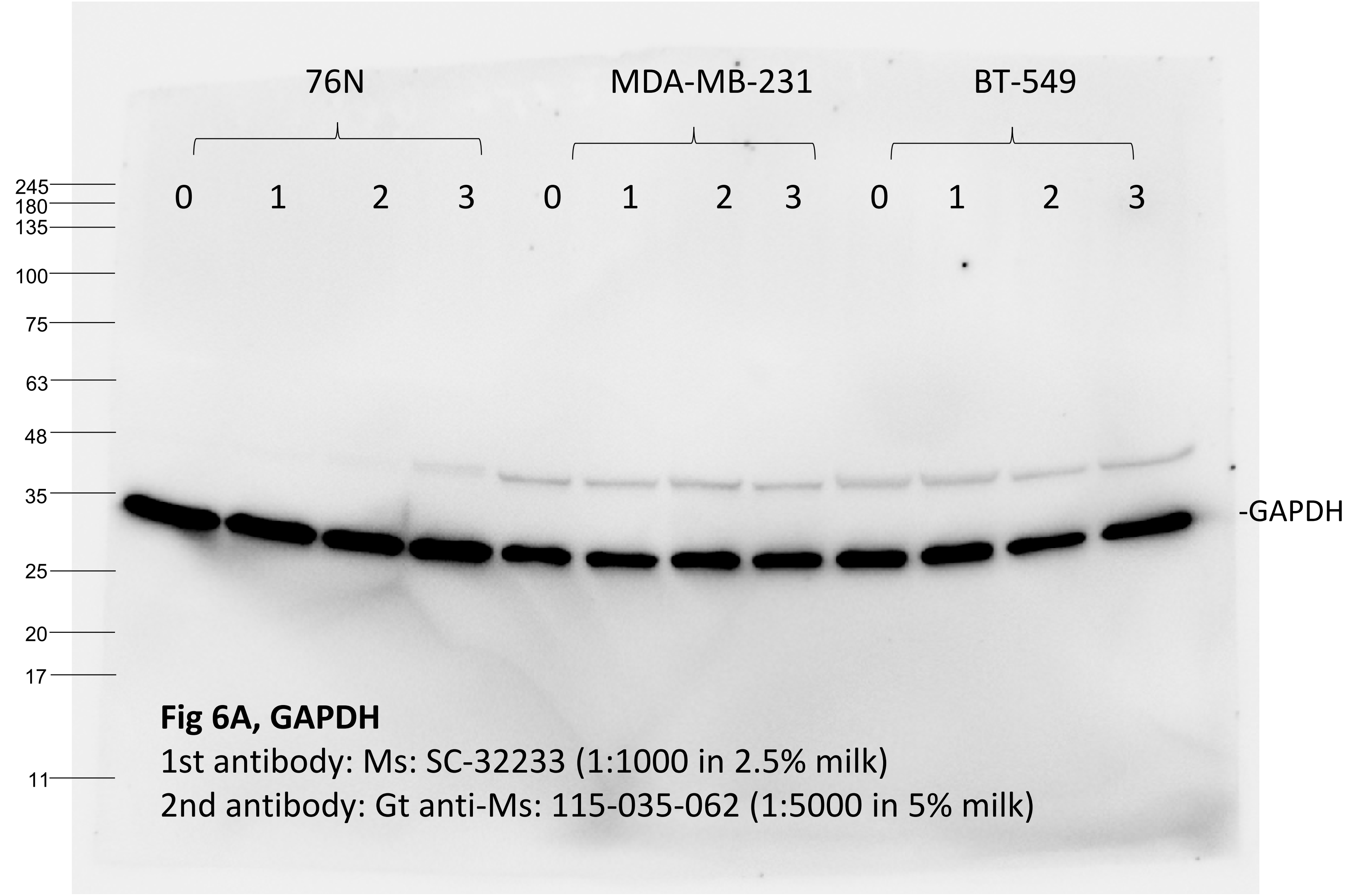

Fig 6A, GAPDH

1st antibody: Ms: SC-32233 (1:1000 in 2.5% milk)

2nd antibody: Gt anti-Ms: 115-035-062 (1:5000 in 5% milk)

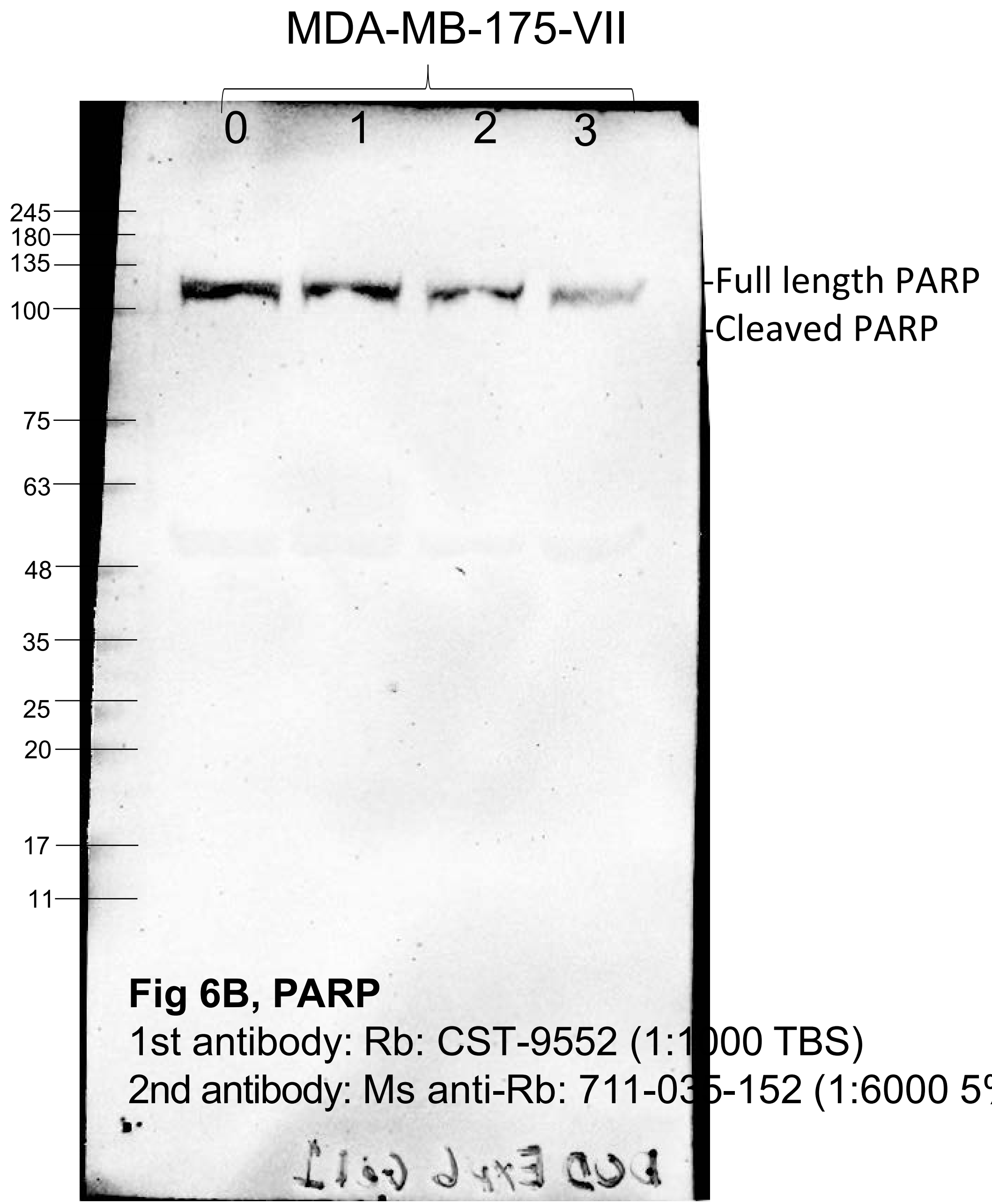

Fig 6B, PARP

1st antibody: Rb: CST-9552 (1:1000 TBS)

2nd antibody: Ms anti-Rb: 711-035-152 (1:6000 5% milk)

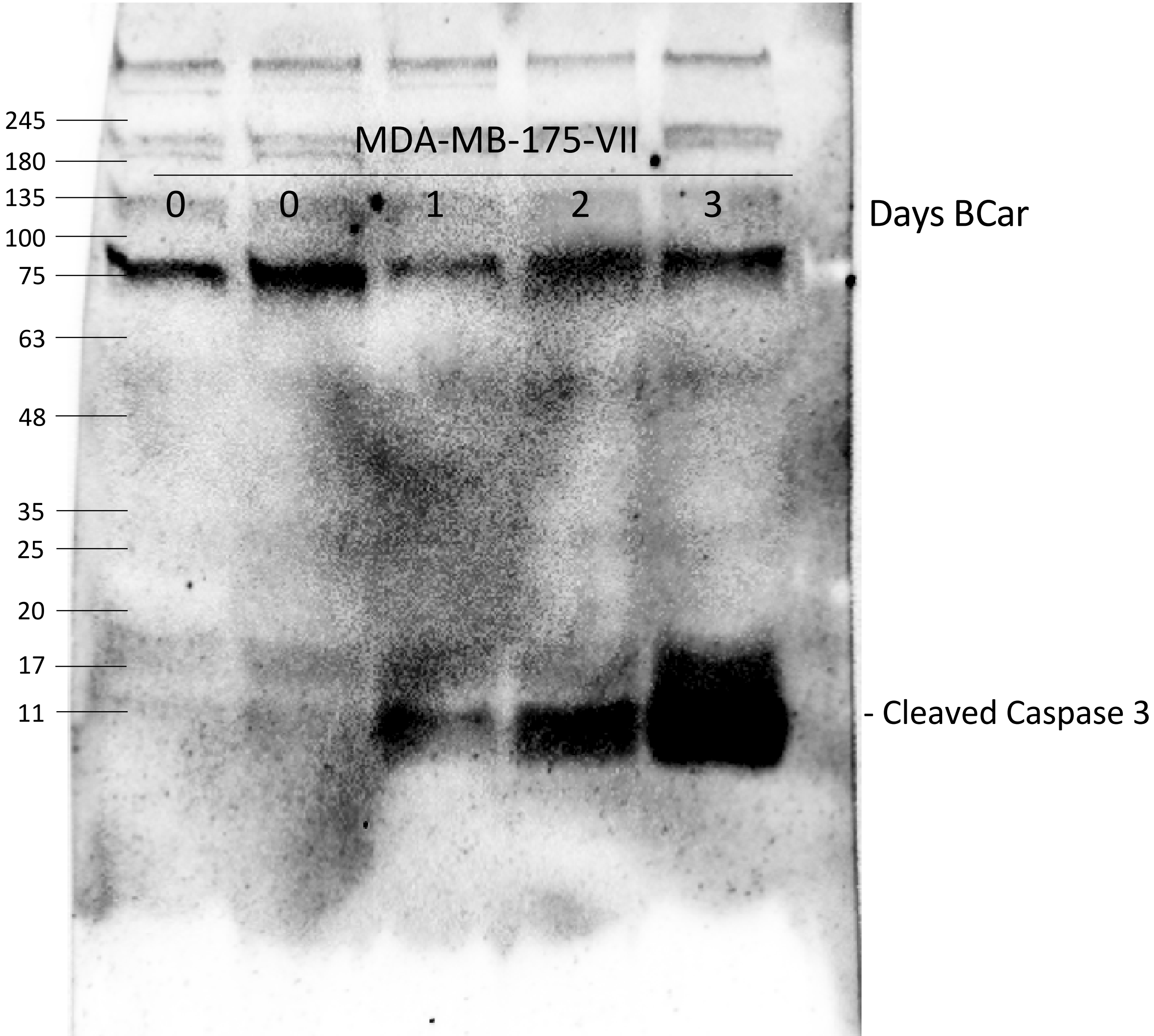

Fig 6B, Cleaved Caspase-3

1st antibody: Rb: CST-9664 (1:1000 in TBS)

2nd antibody: Ms anti-Rb: 711-035-152 (1:6000 in 5% milk)

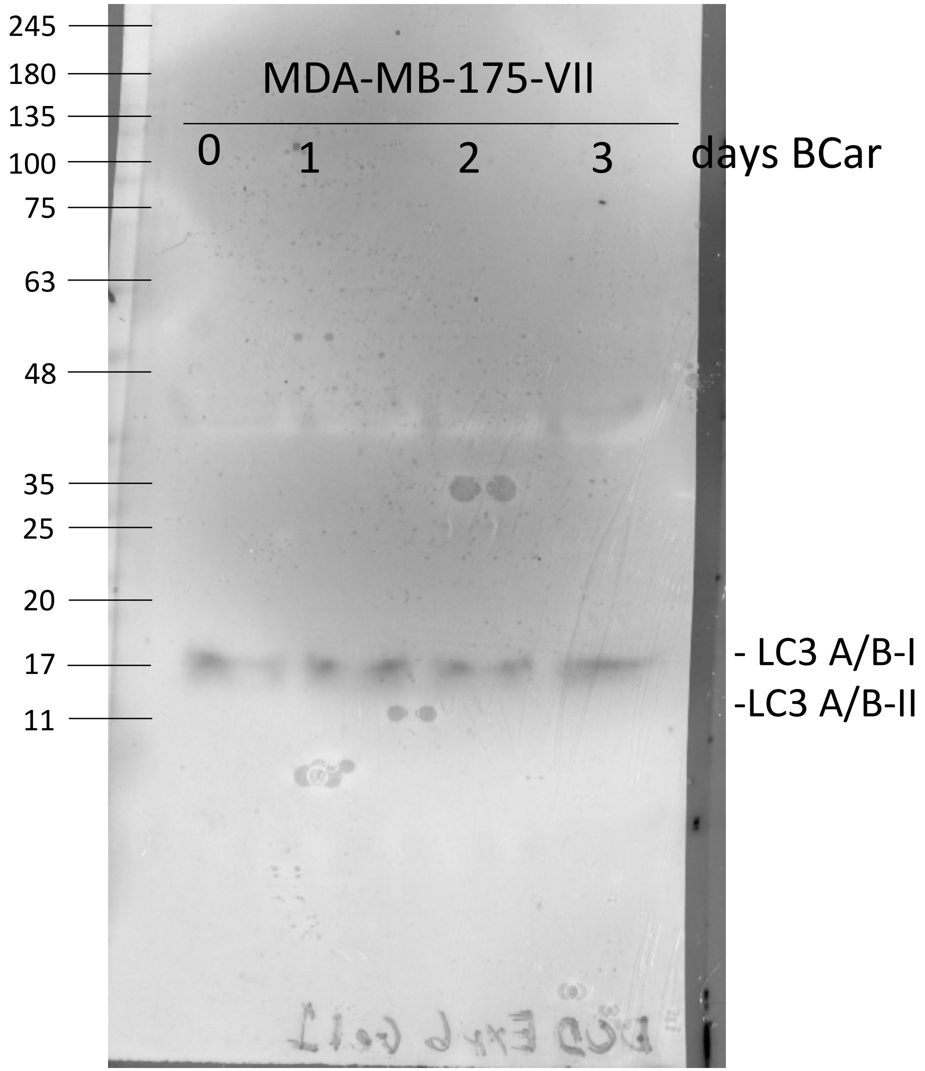

Fig 6B, LC3A/B-I/II

1st antibody: Rb; CST-12741; 1:1000 TBS

2nd antibody: Ms anti-Rb; 711-035-152: 1:6000 5% milk

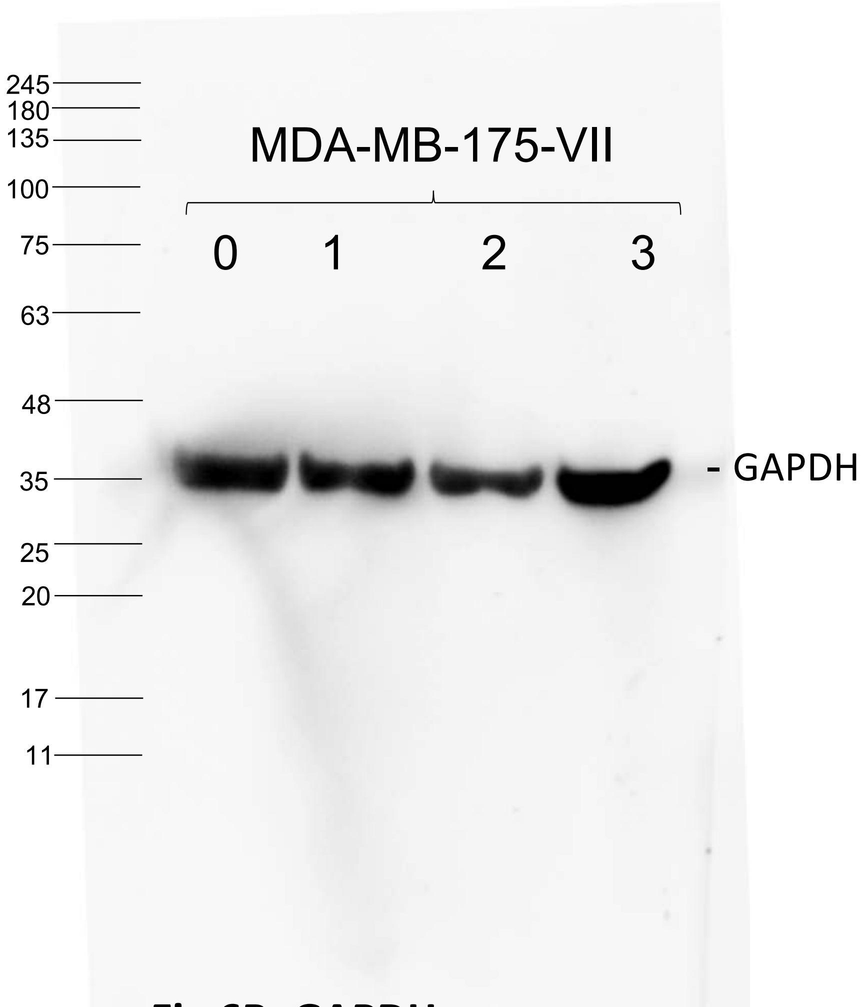

Fig 6B, GAPDH

1st antibody: Ms: SC-32233 (1:1000 in 2.5% milk)

2nd antibody: Gt anti-Ms: 115-035-062 (1:5000 in 5% milk)
