# Supplementary material for: Benzimidazole carbamate induces cytotoxicity in breast cancer cells via two distinct cell death mechanisms
Source: Cell Death Discov. 2023 May 13;9:162. doi: 10.1038/s41420-023-01454-6 (PMC10183037; doi:10.1038/s41420-023-01454-6)
Supplement: Supplementary file 3 — Original Western blots for Figure 7 [file 41420_2023_1454_MOESM3_ESM.pdf]

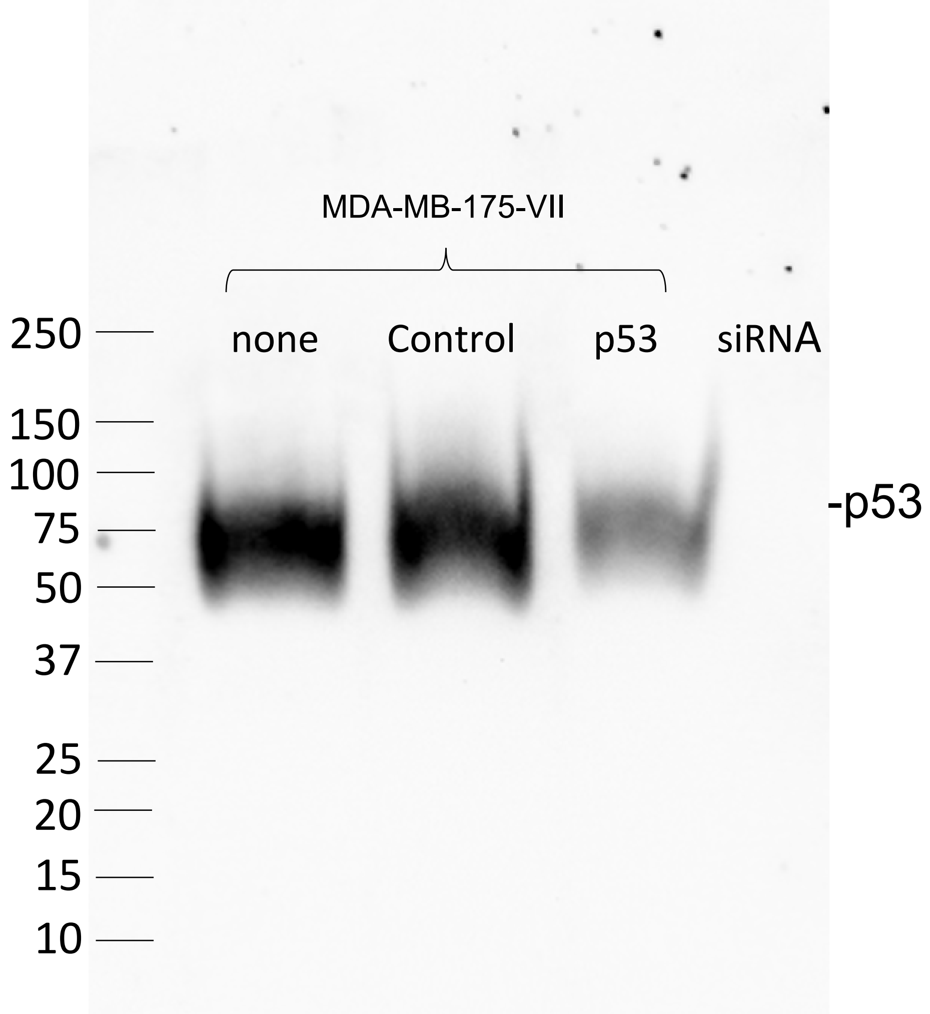

Fig 7A, p53

1st antibody: Ms; SC-126, 1:200 in 5% milk

2nd antibody: Gt anti-Ms; 115-035-062 (Jackson) 1:6000 in 5% milk

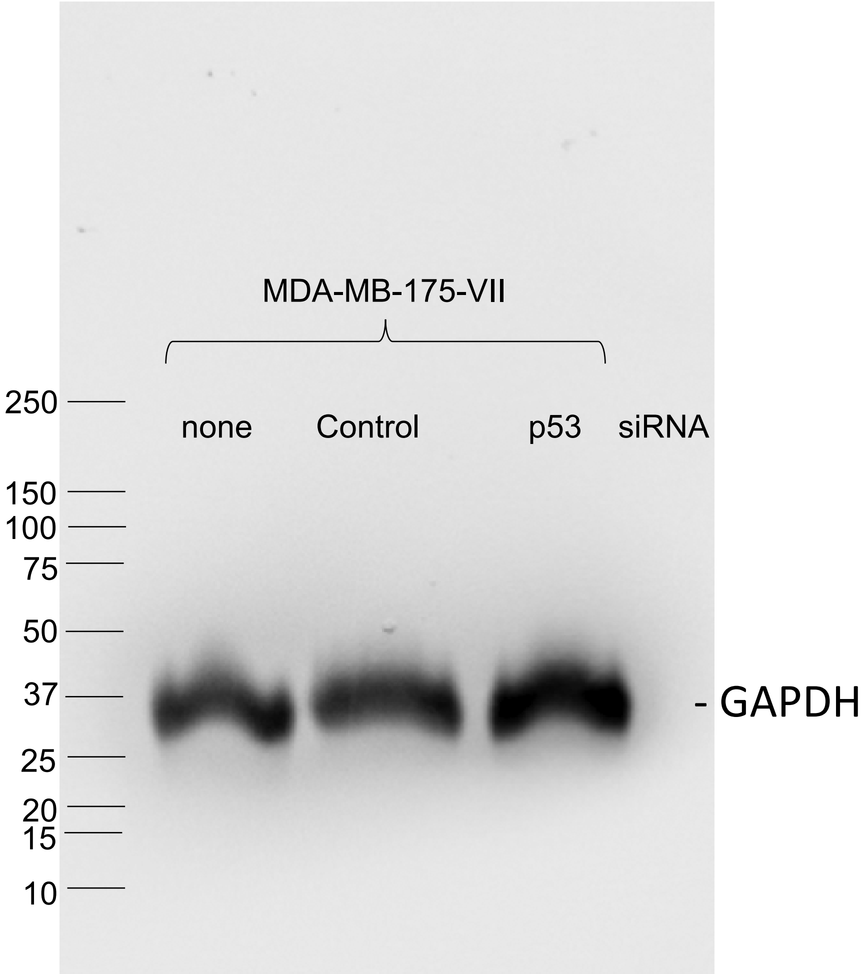

Fig 7A, GAPDH

1st antibody: Ms; Cat# SC-32233, Santa Cruz, 1:1000 in 2.5% milk

2nd antibody: Gt anti-Ms; 115-035-062 (Jackson) 1:6000 in 5% milk

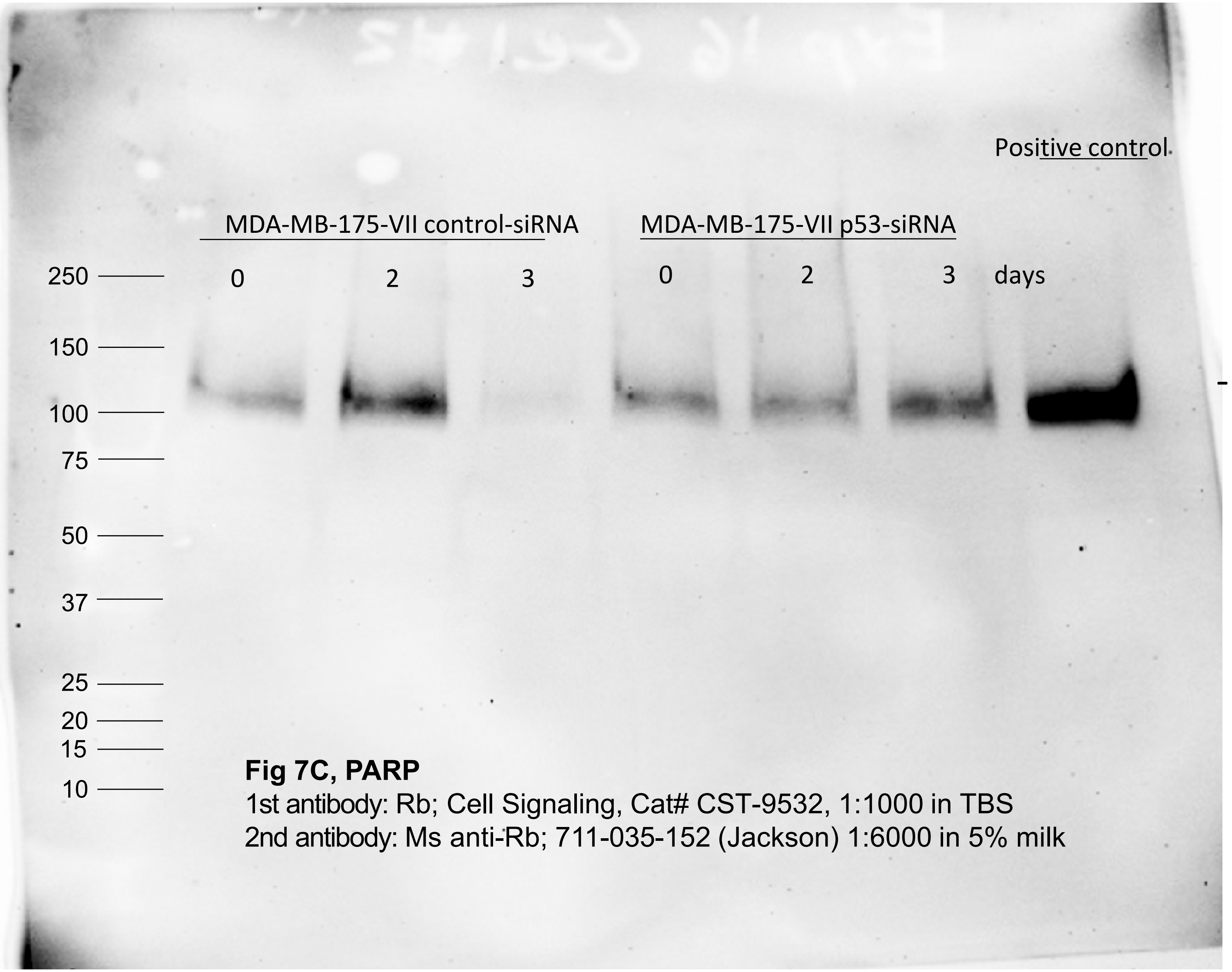

Fig 7C, PARP
1st antibody: Rb; Cell Signaling, Cat# CST-9532, 1:1000 in TBS
2nd antibody: Ms anti-Rb; 711-035-152 (Jackson) 1:6000 in 5% milk

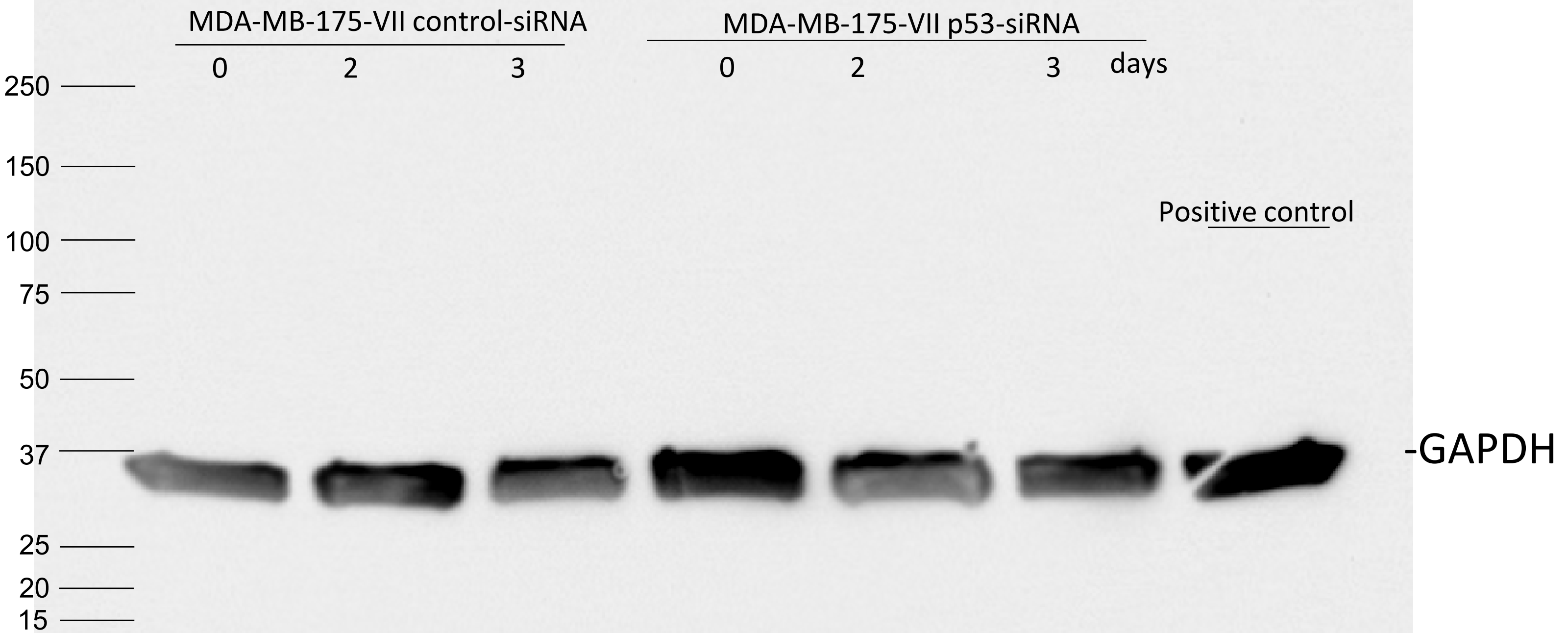

Fig 7C, GAPDH

1st antibody: Ms; SC-32233; 1:1000 5% milk

2nd antibody: Gt anti-Ms; 115-035-062; 1:6000 5% milk
